# Viper Venom Phospholipase A2 Database: The Structural and Functional Anatomy of a Primary Toxin in Envenomation

**DOI:** 10.3390/toxins16020071

**Published:** 2024-02-01

**Authors:** Ana L. Novo de Oliveira, Miguel T. Lacerda, Maria J. Ramos, Pedro A. Fernandes

**Affiliations:** Requimte-Faculty of Sciences, University of Porto, Rua do Campo Alegre s/n, 4169-000 Porto, Portugal; anoliveira@fc.up.pt (A.L.N.d.O.); up201605225@g.uporto.pt (M.T.L.); mjramos@fc.up.pt (M.J.R.)

**Keywords:** snake venom viper, phospholipase A2, toxin, myotoxicity, structure–activity relationship, snakebite envenoming

## Abstract

Viper venom phospholipase A2 enzymes (vvPLA2s) and phospholipase A2-like (PLA2-like) proteins are two of the principal toxins in viper venom that are responsible for the severe myotoxic and neurotoxic effects caused by snakebite envenoming, among other pathologies. As snakebite envenoming is the deadliest neglected tropical disease, a complete understanding of these proteins’ properties and their mechanisms of action is urgently needed. Therefore, we created a database comprising information on the holo-form, cofactor-bound 3D structure of 217 vvPLA2 and PLA2-like proteins in their physiologic environment, as well as 79 membrane-bound viper species from 24 genera, which we have made available to the scientific community to accelerate the development of new anti-snakebite drugs. In addition, the analysis of the sequenced, 3D structure of the database proteins reveals essential aspects of the anatomy of the proteins, their toxicity mechanisms, and the conserved binding site areas that may anchor universal interspecific inhibitors. Moreover, it pinpoints hypotheses for the molecular origin of the myotoxicity of the PLA2-like proteins. Altogether, this study provides an understanding of the diversity of these toxins and how they are conserved, and it indicates how to develop broad, interspecies, efficient small-molecule inhibitors to target the toxin’s many mechanisms of action.

## 1. Introduction

### 1.1. Epidemiology

The World Health Organization recognizes twenty neglected tropical diseases that affect the whole world, but their devastating effects are felt mostly in tropical areas and impoverished communities, where access to hospitals and medicines is limited [1,2]. These neglected tropical diseases can be divided into infectious diseases (19 diseases) and non-infectious diseases, of which there is only one—snakebite envenoming [3,4]. The mortality rate of neglected tropical diseases is ca. 200 thousand people per year, and they cause more than 19 million disabilities annually. In the last decade, the World Health Organization and governmental agencies have carried out several campaigns and investments, significantly reducing the number of infectious neglected tropical diseases. Nonetheless, the impact of snakebite envenoming remains alarming, with 81–138 thousand deaths yearly, the total of which may equal the number of deaths caused by the other 19 neglected tropical diseases altogether [5]. The intricate chemical and bioactive composition of snake venom means that it is challenging [6] to find a cure for snakebite envenoming. The current state-of-the-art treatment involves administering antibodies purified from the plasma of hyper-immunized equines [7,8]. However, it is very costly, and it requires transportation and storage in cold containers, as well as inpatient administration due to frequent anaphylactic reactions [9]. These constraints mean that this therapy is unavailable in the remote regions of resource-poor countries and in poor communities where most snakebites occur [1,7].

In order to move one step closer to solving the problem, significant research focusing on the mechanism of action of venom toxins is required, so as to understand their physiologic activities, for the subsequent development of new, heat-stable, cheap, efficient, and affordable antidotes.

### 1.2. Venom Composition

Snake venoms are complex cocktails comprising tens to hundreds of components, of which >90% (*w*/*w*) are proteins and peptides [10,11,12]. The identity and abundance of components are diverse, and they depend on the snake species, gender, age, habitat, and prey, among other factors. Intraspecific variability is also significant. Venomous snakes belong to three families of front-fanged snakes, the Viperidae (vipers), Elapidae, and Atractaspidae, and a family of rear-fanged venomous snakes, the Colubridae; however, regarding the latter, not all members are venomous [13]. This work focuses primarily on viper venom, as vipers are estimated to be responsible for a substantial proportion of snakebite fatalities [14].

Despite the fact that viper venom is composed of highly diverse elements, three enzymatic toxin families stand out due to their toxicity and abundance: metalloproteinases (vvMPs), secreted phospholipase A2 enzymes (vvPLA2s), and serine proteases (vvSPs) [12]. These families frequently account for ca. 70% of the total venom weight. Other components that are often present, but in smaller amounts, are the C-type lectins, cysteine-rich secretory proteins, and L-amino acid oxidases [15] (for a more complete list, see Ref. [15]). vvMPs and vvPLA2s are present in more than 80% of the ca. 165 known viper venom proteomes [12].

### 1.3. Viper Venom Phospholipase A2 Enzymes

#### 1.3.1. Pathophysiology of vvPLA2s

vvPLA2 is the second most abundant protein family in viper venom on average, surpassed only by vvMPs. It is thus evident that vvPLA2s and vvMPs are prime drug target candidates for treating snakebite envenoming. vvPLA2s play many pathophysiologic roles; regarding the ones found in viper venoms, the most common is myotoxicity [16,17,18,19,20]. Nevertheless, other usual effects caused by vvPLA2s include platelet aggregation, anticoagulation, hemolysis, cytotoxicity, and neurotoxicity [21,22]. They also participate in inflammatory processes and display bactericidal activities [23]. As a whole, viper envenomation produces highly harmful consequences, such as muscle breakdown, neuromuscular paralysis, tissue necrosis and amputations, bleeding disorders, thrombosis, kidney and heart failure, hypovolemic shock, and often death [24,25,26,27], effects for which vvPLA2 is at least partially responsible.

#### 1.3.2. The Structure of vvPLA2

**Figure 1:**
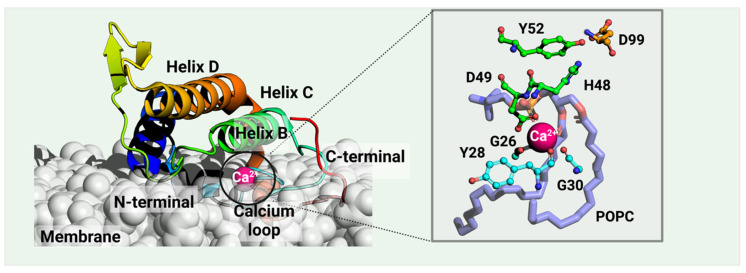
A catalytic vvPLA2 (*Echis carinatus*, PDB ID: 1OZ6 [31], in rainbow) bound on a phospholipid bilayer cell membrane (white) is shown. The Methods section provides the details of the protein–membrane complex modeling. The phospholipid bound to the catalytic cavity is represented by lilac sticks. Residues H48 and D99 compose the catalytic dyad, the latter stabilized by Y52. vvPLA2s need a Ca^2+^ cofactor for catalytic activity, which is heptacoordinated by D49 (double coordination), three peptide carbonyl oxygens of the “calcium loop” (residues G26, Y28, and G30), and the substrate’s phosphate and sn-2 ester bond. Residue numbering follows the standard numbering of Renetseder et al. [32].

vvPLA2s are small, secreted proteins of 13–15 kDa. Together with the human synovial fluid PLA2, they form the PLA2 group IIA [28,29,30]. Within this group, almost all enzymes have seven disulfide bonds, significant sequence identity, and a common fold (Figure 1). Rare exceptions within the viper family include the Gaboon viper (*Bitis gabonica*) and the Rhinoceros viper (*Bitis nasicornis*) vvPLA2s, which form six disulfide bonds and are placed in group IIB.

The vvPLA2 enzymes are further characterized by their isoelectric point. Accordingly, acidic vvPLA2s typically have an isoelectric point of 4.0–5.5 with many acidic, negative residues, and basic vvPLA2s have an isoelectric point of ca. 8.0 with primarily basic and positive protein surface residues. The isoelectric point influences the vvPLA2 toxicity, and as a consequence, basic enzymes are far more toxic than acidic ones. The underlying reason still needs to be clarified but is supposed to lie in the different affinities that the acidic and basic enzymes have for the cell membrane phospholipids and/or for the cell membrane protein receptors.

#### 1.3.3. The Catalytic Activity of vvPLA2

**Figure 2:**
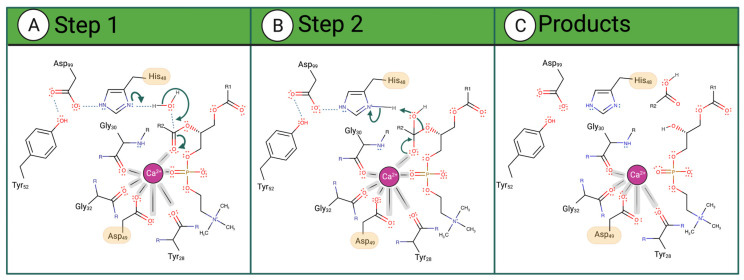
Proposed catalytic single-water mechanism of sPLA2 for a phosphatidylcholine substrate. The reaction occurs in 3 steps: (**A**) His48 deprotonates a water molecule whose hydroxide ion attacks the substrate sn-2 carbon (**B**) The oxyanion collapses, eliminating the phosphocholine substrate that deprotonates His48. (**C**) The fatty acid and the lysophosphatidylcholine are released as products.

vvPLA2 enzymes cleave phospholipids at position sn-2, releasing fatty acids and lysophosphatidic acids. The corresponding mechanism of this hydrolysis reaction is only partially understood at the atomic level, occurring always in the presence of a Ca^2+^ cofactor. The latter is coordinated to Asp49 (bidentate), three carbonyl groups belonging to both a glycine and a tyrosine or phenylalanine residues located in the Ca^2+^-binding loop, and the substrate’s phosphate and sn-2 carbonyl groups (or two water molecules when in the unbound state). According to the postulated mechanisms, His48 deprotonates one water molecule, either directly (“single-water mechanism” hypothesis) or through a second bridging water molecule (“assisted water mechanism” hypothesis), generating a hydroxide ion that attacks the sn-2 carbon of the phospholipid (Figure 2A). The hydrogen bonding of His48 to the carboxylate of Asp99 stabilizes the positive form of His48, facilitating water deprotonation. Whether or not the nucleophilic water molecule is Ca^2+^-bound is unclear. Anyhow, the Ca^2+^ stabilizes the reaction’s transition state by coordinating the oxyanion generated at the sn-2 carbonyl site (and eventually the hydroxide nucleophile), subsequently decaying into a tetrahedral intermediate (Figure 2B). Following the ester bond cleavage, the hydrolyzed products—fatty acid and lysophospholipid—are released (Figure 2C). No evidence exists for protein conformational changes during the cycle [33].

#### 1.3.4. The vvPLA2 Protein–Membrane Interface

Monomeric and dimeric vvPLA2s are the most common quaternary structures found via X-ray crystallography [19,34,35,36], and they are supposed to be the most common in solution too.

Two different dimeric quaternary structures have been found in cristallo, named “conventional” and “alternative” [37] (Figure S1). Nevertheless, we will refer to them here as “extended” and “compact” dimers, respectively, as this nomenclature better reflects the nature of their quaternary structure, and the fact that the buried area of these two structures is very different. The extended dimer of a PLA2-like protein (i.e., a non-enzymatic homolog of the vvPLA2 enzymes, as discussed in Section 1.3.6), from, e.g., *B. pauloensis*, buries an area of 1022 Å^2^ (Ref. [38]), and it has few intermonomer contacts, which may turn the dimerization in water less robust. Nevertheless, it has phospholipid binding sites open to the solvent. The compact conformation of the same enzyme achieves a larger buried area of 1491 Å^2^ (Ref. [39]) and establishes many more intermonomer contacts than the extended conformation. It seems more stable in water, even though each monomer occludes the binding site of the other (more examples in Table S1). Thus, a conformational change or dissociation may be needed to expose the binding site of a compact dimer to the cell membrane (Figure S2).

vvPLA2s and similar human pancreatic PLA2s are believed to bind the cell membrane as monomers in most cases [40]. Therefore, if a PLA2 forms dimers in solution, dimer dissociation should precede or happen concomitantly with membrane binding. vvPLA2s have more affinity for membrane regions richer in negative than zwitterionic phospholipids, particularly the most toxic basic isoforms. Insights from an anion-assisted dimer of pancreatic porcine PLA2 [41,42] estimate that PLA2 buries 30–40 phospholipids, which form salt bridges with the PLA2 basic residues [43]. Acidic vvPLA2s are generally catalytically more active in vitro but less toxic in vivo than the basic isoforms [44]. In contrast, most basic vvPLA2s induce several toxic effects [17,26,37,45,46].

#### 1.3.5. The Role of the N-Terminal Region for Membrane Binding and Enzymatic Activity

vvPLA2s contain an α-helical N-terminal region believed to be implicated in critical functions, such as membrane binding, and vital structural areas, such as the substrate-binding hydrophobic channel. X-ray structures of PLA2s from the Indian cobra (*Naja naja*) [47] and the European bee (*Apis mellifera*) [48] venoms and from human [49] and pig (*sus scrofa*) [50] secretory PLA2s, solved in the presence of transition-state analogs, showed the N-terminal region interacting with the phospholipid substrate (Figure S3). The invariant residues Leu2, Phe5, and Ile9 delimitate the substrate-binding cavity on one side. The contribution of the N-terminal region to the catalytic activity has been extensively studied in mammalian PLA2s, where the deletion of 8–10 residues of the N-terminus resulted in a nearly complete loss of enzyme activity [51,52]. Moreover, deleting the ten N-terminal residues made the enzyme bind to membranes with lower affinity and random, non-specific orientations [53]. Such studies underline the importance of the N-terminal region in regulating the PLA2 function by determining the strength of membrane binding and the productive orientation of PLA2 at the membrane surface and in contributing to possible structural changes in the enzyme during interfacial activation [54,55]. However, few studies regarding this topic were performed with vvPLA2. Nevertheless, studies involving a vvPLA2 from *Crotalus atrox* confirmed that deleting the ten N-terminal residues results in a loss of enzymatic activity as well as the dimeric structure of the native enzyme [56].

#### 1.3.6. The PLA2-Like Proteins and Their Myotoxic C-Terminal Region

**Figure 3:**
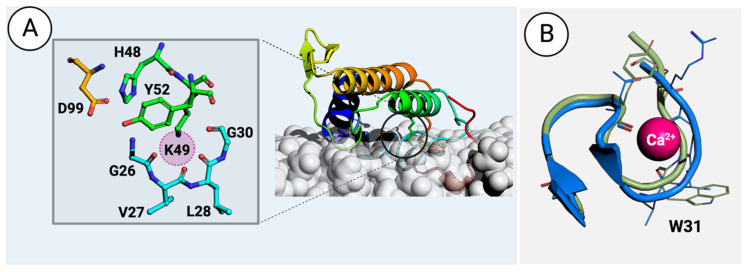
(**A**) A PLA2-like protein from the venom of *Bothrops asper* (PDB ID: 1Y4L [57]) interacts with a phospholipid bilayer membrane. The details of modeling the protein–membrane complex are given in the Methods section. The inset on the left shows the residues surrounding and stabilizing the H48K49C50C51 motif. The sphere in magenta highlights the position occupied by the calcium atom in vvPLA2. (**B**) Superimposition of the vvPLA2 Ca^2+^ loop (green) on the equivalent loop region in the PLA2-like proteins (blue). The small difference in the position of the loop is sufficient to affect the binding of the Ca^2+^ ion.

There are two types of PLA2 proteins in viper venom: the previously discussed Ca^2+^-dependent catalytically active enzymes (vvPLA2) and the non-enzymatic PLA2 proteins (Figure 3). The latter are named PLA2-homologs, Lys-49 PLA2, or PLA2-like proteins (the last designation will be used here) and are exclusively found in viper venoms as they have diverged from the former during evolution.

The main differences found between vvPLA2 and PLA2-like proteins are as follows:

i. The residue at position 49, which can be Asp in the enzymes and Lys or, more rarely, Ser, Asn, Gln, or Arg, in the PLA2-like proteins);
ii. The active site Ca^2+^ cofactor, which is only present in vvPLA2;
iii. The sequence and fold of the Ca^2+^-binding loop;
iv. The sequence of the C-terminal region [43]. Viper venoms generally contain a mixture of vvPLA2s and PLA2-like isoforms, whose synergistic action determines their function and pathological effects [58].

PLA2-like proteins have been extensively studied for their ability to disrupt the cell membrane, causing myotoxicity [16,58,59,60,61,62,63,64]. A large body of studies recently reviewed in Ref. [57] identifies the C-terminal region (residues 105–117) in the mature *Bothrops asper* myotoxin II, Uniprot ID: P24605, or equivalently residues 115–129 when using the standard numbering (i.e., a common residue numbering for mammalian pancreatic and venom PLA2 enzymes [32]), composed primarily of cationic, hydrophobic, and aromatic residues, as the primary determinant for myotoxicity. Moreover, the same region is involved in hyperalgesia and inflammation. In several instances, peptides with the 115–129 sequence retain, partly or wholly, the bioactivity of the complete enzyme [60]. However, given the differences between the bioactivity of the total enzyme and the 115–129 peptide, studies suggest that the C-terminal region is central but not solely responsible for PLA2 toxicity, with other residues, such as K20, K36, and K38, also being crucial for the effect [16,45,64,65]. Like vvPLA2, PLA2-like proteins induce local membrane perturbations, allowing Ca^2+^, K^+^, and ATP to be internalized through membrane crossing, leading to extreme cytotoxic events [66,67].

#### 1.3.7. Evaluation of vvPLA2 Druggability

Researchers are developing small-molecule drug treatments against snakebite envenoming to overcome the limitations of the antibody-based treatment [68,69,70]. The vvPLA2s are a primary drug target because they are expressed in more than 90% of viper species [10,11,12] and are associated with drastic pathologic processes [21,71,72]. Moreover, it is known that competitive enzymatic inhibitors such as varespladib [69,73,74,75,76,77,78,79] also inhibit PLA2-like myotoxicity [69,74,80]. Given the conservation of the binding pocket, the design of an interspecific vvPLA2 inhibitor is a possibility that will be explored in this work.

#### 1.3.8. Important Open Questions about vvPLA2 and PLA2-like Proteins

Some of the central questions out of the many unexplained aspects of this important family of proteins are as follows:

i. How extensively are the 3D structures and sequences of vvPLA2 and PLA2-like proteins conserved?
ii. How conserved is the vvPLA2 orientation and position in the cell membrane?
iii. Is it possible to design a universal anti-PLA2 drug with anti-envenomation activity for humans?
iv. What are the significant similarities and differences in the N-terminal region, and how do they affect membrane binding?
v. What is the molecular mechanism through which PLA2-like proteins exert myotoxicity?

These are the questions that we are looking into in the present study. Question (i) is addressed in Section 2.2; question (ii) is tackled in Section 2.3; question (iii) is dealt with in Section 2.4; question (iv) is answered in Section 2.5; and, finally, the challenging question (v) is taken up in Section 2.6, where insights into a possible explanation are provided. We performed extensive bioinformatics analyses and molecular modeling on PLA2 sequences and structures deposited in the UniProt Database to answer these questions and relate their structural traits with known bioactivities. Consequently, we complement the X-ray structures with high-accuracy computational 3D structures of a vast number (217) of vvPLA2 and PLA2-like proteins. The results provide a new understanding of the molecular-level determinants behind the vvPLA2 and PLA2-like pharmacological activities, which can be exploited easily for drug discovery against snakebite envenoming.

## 2. Results and Discussion

**Figure 4:**
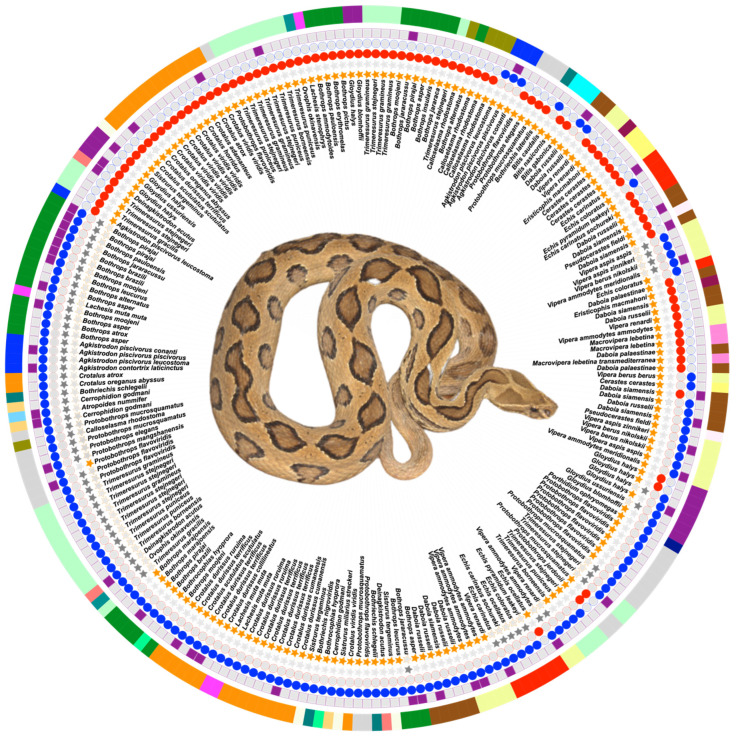
Summary of the properties of the 217 structures from 79 species and 24 genera analyzed in this work. vvPLA2 entries are identified with an orange star; PLA2-like proteins are identified with a grey star. Acidic proteins are marked with red circles, while basic entries are marked with blue circles. The entries having X-ray structures are marked with purple squares. The outermost marking on the image represents the genus, and each genus has been assigned a distinct color. In the center is shown a photograph of *Daboia russelii*, one of the deadliest vipers in the world. Photo courtesy of Gowri Shankar, Kalinga Centre for Rainforest Ecology, India.

We retrieved all the PLA2 UniProt [81,82] entries with the status “Reviewed” from the Viperidae family up to May 2023, resulting in 346 sequences. We excluded incomplete sequences (84 entries), phospholipase A2 inhibitors (38 entries), and sequences that had ambiguous residues (7 entries), resulting in 217 different PLA2 entries from 24 genera and 79 species with a wide geographic distribution. The 217 entries were divided into vvPLA2s (160 sequences), 89 acidic and 71 basic isoforms, and PLA2-like proteins (57 sequences of basic isoforms). Figure 4 summarizes the composition of the database.

### 2.1. A Validated Database of vvPLA2 and PLA2-like Tridimensional Structures

To determine the accuracy of the homology modeling protocol, we compared the constructed models with the corresponding X-ray structures for 12 species available in the PDB. Three quality criteria were used: the correct number of disulfide bonds and the average and maximum RMSD values in relation to the X-ray structure. The results indicate that the models built from Good-Quality or High-Quality templates have RMSD values that, on average, differ by 1.0 Å and 0.8 Å from the X-ray structures, which is close to the experimental error. Furthermore, the maximum RMSD of any system built from Good-Quality and High-Quality templates is 1.3 Å and 1.1 Å, emphasizing the robustness of the modeling (Table 1, Tables S2 and S3). Medium-Quality templates generate models with an average RMSD value of 1.6 Å in relation to the X-ray structures, which nonetheless is a very reasonable result.

In sum, the protocol used here allows for obtaining PLA2 structures with a very satisfactory quality when built from Medium-Quality templates or an accuracy within the error of many experimental X-ray experiments when built from Good-Quality and High-Quality templates.

Furthermore, we have compared the accuracy of our models with the AlphaFold structures deposited in the UniProt database. In general, the Ca^2+^-binding loop displays two principal conformations: open and closed. The open conformation exists in the apoenzyme, and the closed in the holoenzyme. As AlphaFold predicts the apoenzyme structure, it mainly generates enzyme models in the open conformation. However, this differs from the conformation we are interested in, which we have modeled: the reactive holoenzymes with Ca^2+^ bound and with a closed loop. Therefore, there is an intrinsic difference between our homology and AlphaFold models. Apart from this aspect, the RMSDs show that our structures and those of AlphaFold are similar, with minimum and maximum RMSDs of 0.4 Å and 0.7 Å (Table S4).

After successfully validating the high accuracy of the homology modeling, the protocol was applied to all the sequences that lacked tridimensional information and were present in our curated sequence database, generating a new database with the 3D structures for the 217 vvPLA2 and PLA2-like proteins. The complete database is available for download as a package of Supplementary Materials.

### 2.2. PLA2 Key Structural Features

vvPLA2s share a significant sequence identity (*ca*. 60% on average) and a global fold that allows an almost perfect superposition of all generated models.

**Figure 5:**
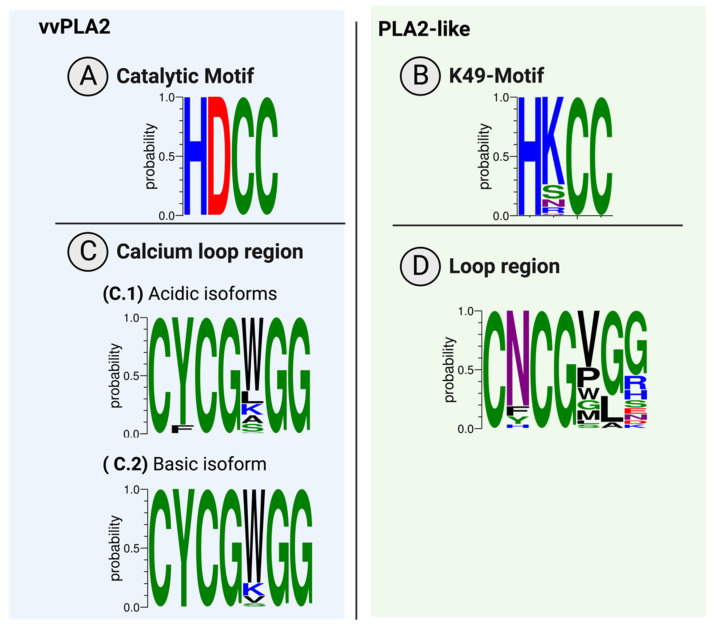
Conservation of (**A**) the catalytic motif and (**B**) the equivalent region in the PLA2-like proteins and (**C**) the Ca^2+^-binding loop in acidic and basic vvPLA2s and (**D**) the equivalent region in the PLA2-like proteins. The sequence conservation logos were created with WebLogo3 [84,85].

**The active site and Ca^2+^-binding loop:** The vvPLA2s have an active site that can be seen as a variant of the serine esterase family where the serine is replaced by a water molecule (as in the deacylation step of serine esterases). The catalytic dyad (His48/Asp99) is strictly conserved in vvPLA2s (Figure 5A), and the Ca^2+^ cofactor replaces the classical hydrogen-bond-based oxyanion hole of serine esterases. The Ca^2+^-coordinating Asp49 is replaced by a Lys residue in 68% of the database PLA2-like proteins (Figure 5B) and by a Ser, Asn, Gln, or Arg in the remaining cases. The catalytic machinery is essentially preserved in the PLA2-like proteins, supporting the hypothesis that the latter can catalyze the breaking of the sn-2 bond but become inactivated due to inefficient product release [33].

The region of the Ca^2+^ loop that encircles the catalytic cavity (C_27_YCGXGG_33_, X= W/K/L/A vs. CNCGX_a_X_b_X_c,33_, X_a_ = V/P/W/G/M, X_b_ = G/L/A, X_c_ = G, R, S, E, N, D, K, Figure 5C,D) presents a significant difference between the vvPLA2 and PLA2-like proteins, which is not surprising, given that the PLA2-like proteins do not bind Ca^2+^: four of the vvPLA2s’ highly conserved residues in this region are not conserved in the PLA2-like proteins. The conservation of the Ca^2+^ loop is much greater within the vvPLA2s than within the PLA2-like proteins, reflecting the less relevant role of the loop in the latter. An Asn residue replaces the vvPLA2 Tyr28 in 75% of the PLA2-like proteins of the database. The Trp31of vvPLA2 is replaced in 90% of the PLA2-like proteins by smaller residues such as Val (44%), Pro (20%), and Gly (10%). Pro and Gly are amino acids with unique properties due to the very short sidechain of Gly and the cyclized chain of Pro, which often lead to unusual backbone conformations. The vvPLA2 residues Gly30 and Gly32 coordinate the Ca^2+^ cofactor with the backbone carbonyl groups. These residues are strictly conserved in all vvPLA2s. However, in PLA2-like sequences, although the Gly30 residue is strictly conserved, Gly32 is conserved only in ~75% of the database sequences. Finally, Gly33 and Gly32 are essential to keep the loop in its catalytic conformation. The conservation of these residues is reduced in the PLA2-like proteins of the database, and larger hydrophobic residues replace several entries (67%) at position 32 or 33; the last even contains charged amino acids (41%).

Other essential residues for vvPLA2 are Tyr52 and Asp99, which define the His48 conformation through an H-bond network in the modeled and X-ray structures and stabilize the positive form of His48 when the latter deprotonates the nucleophilic water molecule (Figure 1). Both are highly conserved among the vvPLA2 in the database; the only exception is an Asp99Arg mutation in the acidic vvPLA2 from *B. pauloensis* (UniProt entry D0UGJ0) [83].

The two active site cysteines—Cys27 and Cys29—play a vital role in stabilizing the Ca^2+^-binding loop conformation because they form two strictly conserved disulfide bonds with the Cys114 at the C-terminal region and Cys45 in the adjacent helix. Tyr28 is also highly conserved. This residue is replaced by phenylalanine in all acidic vvPLA2s of the *Trimeresurus* genus and the *B. pictus* vvPLA2 (22 structures).

**Other relevant structural aspects:** Another exciting structural characteristic is the significant conservation of Trp31 in the Ca^2+^-binding loop (63% in acidic and 80% in basic vvPLA2s). Tryptophan is a voluminous residue often found buried inside the cell membrane. Thus, this residue may facilitate the anchoring or penetration of the vvPLA2 into the membrane, smoothing the access of the phospholipid substrate to the catalytic cavity. Furthermore, Trp31 is replaced by a Lys residue in ca. 10% of the database sequences. The latter is positively charged and thus can make salt bridges with the negative phospholipid phosphates, also helping to anchor the protein to the membrane.

Other residues found at this position in the acidic vvPLA2 are Leu (12%), Ala (11%), and Ser (7%). In the case of the basic vvPLA2, Trp31 is also replaced by Val (5%) and Ser (2%). In conclusion, the residue at position 31 either has tryptophan or another hydrophobic residue. The exception is Ser, which appears in rare cases. This suggests that the residue at position 31 may be buried deep within the membrane, beyond the polar headgroups, and may serve as an anchor in the hydrophobic part of the membrane.

Interestingly, Trp31 is always replaced in the PLA2-like proteins (Asn in >80% of cases), meaning that it is not needed for anchoring to the membrane in the latter or that it disturbs the mechanism of membrane destabilization through which its toxicity is manifested. Therefore, other membrane-docking residues should be dominant in PLA2-like proteins. A significant body of studies consistently points out that PLA2-like proteins dock with the membrane through several Lys/Arg residues absent in vvPLA2s. This subject will be discussed in detail in Section 2.3.

### 2.3. The vvPLA2 and PLA2-like i-Face

**Figure 6:**
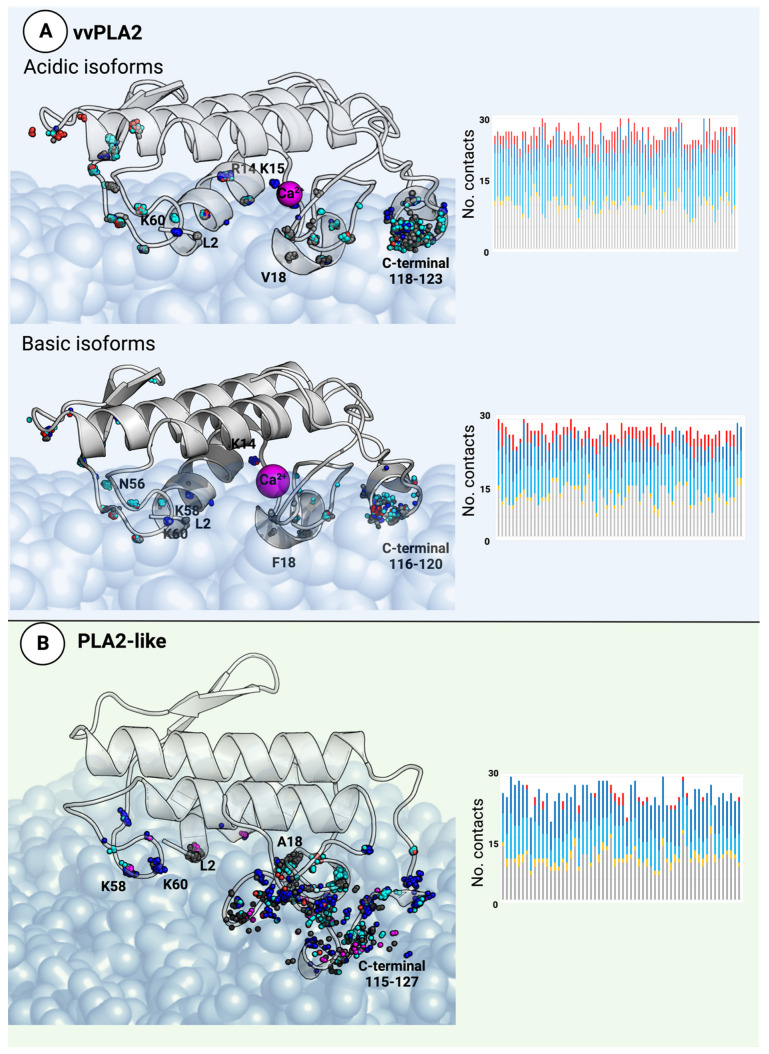
Left, molecular models of the vvPLA2 enzymes (**A**) and PLA2-like proteins (**B**) bound to a phospholipid bilayer. The protein–membrane models were built for all proteins in the database. The residues within 3 Å of the phospholipids are marked as spheres. The spheres’ positions represent the atomic coordinates of the Cα atoms. The polar residues are colored in cyan, the cationic residues in blue, the anionic residues in red, and the non-polar residues in gray. This scheme color is repeated throughout this manuscript. The residues involved in the most conserved interactions are labeled according to the *B. asper* PLA2 isoforms. Right, a stacked bar graph of the protein–membrane interactions shows that the number of interactions of each type is quite similar across all proteins. Each bar corresponds to a single dataset protein and illustrates its number of negative (red), positive (dark blue), polar (light blue), non-polar (gray), and aromatic (yellow) interactions. The identity of the protein that corresponds to each bar is not given in the figure for simplicity but can be found in Table S6.

The protein–membrane interface region (i-face) is defined by the nature of the residues that directly interact with the phospholipid bilayer. Experiments on bovine and porcine pancreatic enzymes indicate that PLA2 enzymes have a higher affinity for anionic rather than neutral vesicles [44,86,87,88,89]. Subsequently, it was suggested that human, porcine, and snake venom PLA2 enzymes need at least 15% negatively charged phospholipids to bind the membrane [90]. The structures of the proteins bound to the cell membranes modeled here show that the i-face is formed by the Leu2 and Gln4 (or Glu4) residues located at the N-terminus; residues 18–23 (predominantly hydrophobic), polar residues at position 58, position 59, or both (which are replaced by charged amino acids in 20% of the analyzed structures of basic vvPLA2s); a highly conserved Lys61 residue (>91%); residues 67–69 (two polar residues and one aromatic residue); and residues 105–113 at the C-terminus (mostly positive and aromatic residues, with a composition to be discussed in detail in Section 2.6). Figure 6A illustrates the protein–membrane interactions. vvPLA2 basic isoforms have several aromatic and lysine residues on the i-face, which interact with the negative phospholipids and thus justify the higher affinity of this subgroup towards the membrane when compared to their acidic counterparts (Figure 6A). The generated 3D protein–membrane structures show that the i-face of vvPLA2 is constituted by several regions spread along the protein surface. Effectively, many residues identified on the vvPLA2 i-face were consistent with those belonging to the i-face of the bee venom PLA2 in an anionic membrane deduced with the help of electron paramagnetic resonance spectroscopy [91] and the i-face of pancreatic porcine PLA2 deduced from an X-ray structure [92]. Moreover, mutagenesis studies performed on the pancreatic PLA2 demonstrated that the N-terminal region and the region comprising residues 56–73 play an essential role in the binding interface [93].

In contrast, no direct experimental evidence has been gathered to determine the i-face of PLA2-like proteins. Nevertheless, the binding position of the PLA2-like proteins to the membrane predicted by us using the PPM server [94] is very similar to that of the vvPLA2 determined experimentally, which makes total sense for the following reasons:

i. The fold of the PLA2 enzymes and the PLA2-like proteins is the same. There are only two large flat surfaces on both, which are able to interact with the flat membrane, corresponding to the “bottom” vvPLA2 i-face and the opposite “top” face made of the two parallel alpha-helices and the beta wing. The remaining faces are very narrow, as the proteins and enzymes have a flat disk shape.
ii. The bottom i-face surface is charged with several positive residues in the PLA2-like proteins, making the interactions with negative membrane phosphates altogether very favorable and certainly more favorable than the “top” face.
iii. X-ray structures show that the PLA2-like proteins bind fatty acids in the binding cleft (i.e., the cleft analogous to the active site of vvPLA2s). Examples include PDB IDs 6B83 [95], 6B81 [95], 6B80 [95], and 1XXS [96] (*B. moojeni*); 1S8G [97] (*Agkistrodon contortrix laticinctus*); and 2QHD [98] (*E. carinatus,* with a Ser residue at position 49). The 1XXS structure has two fatty acids in the binding cleft, resembling a complete phospholipid. The binding of PLA2-like proteins to the membrane through the same i-face as vvPLA2s allows for straightforward uptake of membrane fatty acids, as the binding pocket is unobstructed and lies at the top of the cell membrane (Figure 3). Other binding positions favor easy fatty acid capture from the membrane.

Considering these arguments, it is highly likely that the PLA2-like proteins bind to the membrane using the same i-face as their homologous PLA2 enzymes do. In addition, it is also likely, although not mandatory, that they bind as monomers, as their homologous PLA2 enzymes do, due to the necessary opening of the compact dimers to expose the binding cleft to the membrane. Nevertheless, if the binding is completed with the PLA2-proteins dimerized as extended dimers, the i-face is the same to allow for the uptake of fatty acids and phospholipids (Figure S5). Thus, a monomeric model is adequate for the present study as it covers both the monomeric and extended-dimeric associations.

In the PLA2-like proteins, the contacts are less spread and more localized in three regions: the N-terminal region, the 23–33 segment (corresponding to the calcium loop segment in vvPLA2 and adjacent residues), and the C-terminal region (Figure 6B). Moreover, it is evident from Figure 6B that the number of membrane contacts of PLA2-like proteins is larger than that in the vvPLA2 enzymes. Positively charged residues largely contribute to anchoring the PLA2-like proteins to the membrane, followed by the aromatic residues. However, we also found several polar and hydrophobic contacts in PLA2-like proteins in a much more significant proportion than observed on the catalytic enzymes. The importance of the hydrophobic residues located at the C-terminal region for membrane binding was previously described for a *B. asper* PLA2-like protein [25], and our results strongly support their importance for many other PLA2-like isoforms from other species.

In addition, the nature of the contacts is well (although not strictly) conserved. This can be seen in Figure 6, where spheres of the same color aggregate together, meaning that residues that provide interactions of the same nature are mostly found in similar spatial locations. This is even clearer if the aromatic and hydrophobic residues (gray and magenta spheres) are grouped, as they provide similar, albeit not equivalent, intermolecular interactions.

### 2.4. The Ligand-Binding Cavity

The only cavity in vvPLA2s is surrounded on one side by the tip of the hydrophobic tunnel that drives the substrate into the active site and, on the other side, by the catalytic motif and the Ca^2+^ cofactor and loop. Four structures of PLA2 enzymes bound to transition-state analogs—1POB (*Naja atra* venom) [47,48], 1POE (*Homo sapiens* synovial fluid) [49], 1POC (*Apis mellifera* venom) [48], and 5P2P (*Sus scrofa* pancreatic) [50]—indicate that the substrate occupies this cavity (Figure S3). In addition, for a reason far from obvious, the myotoxicity of PLA2-like proteins is also inhibited by ligands bound in the protein active-site-like cavity. We hypothesize that phospholipid or fatty acid binding is essential for proper membrane binding in the PLA2-like proteins. Thus, the latter is inhibited by the vvPLA2 competitive inhibitors as well.

**Figure 7:**
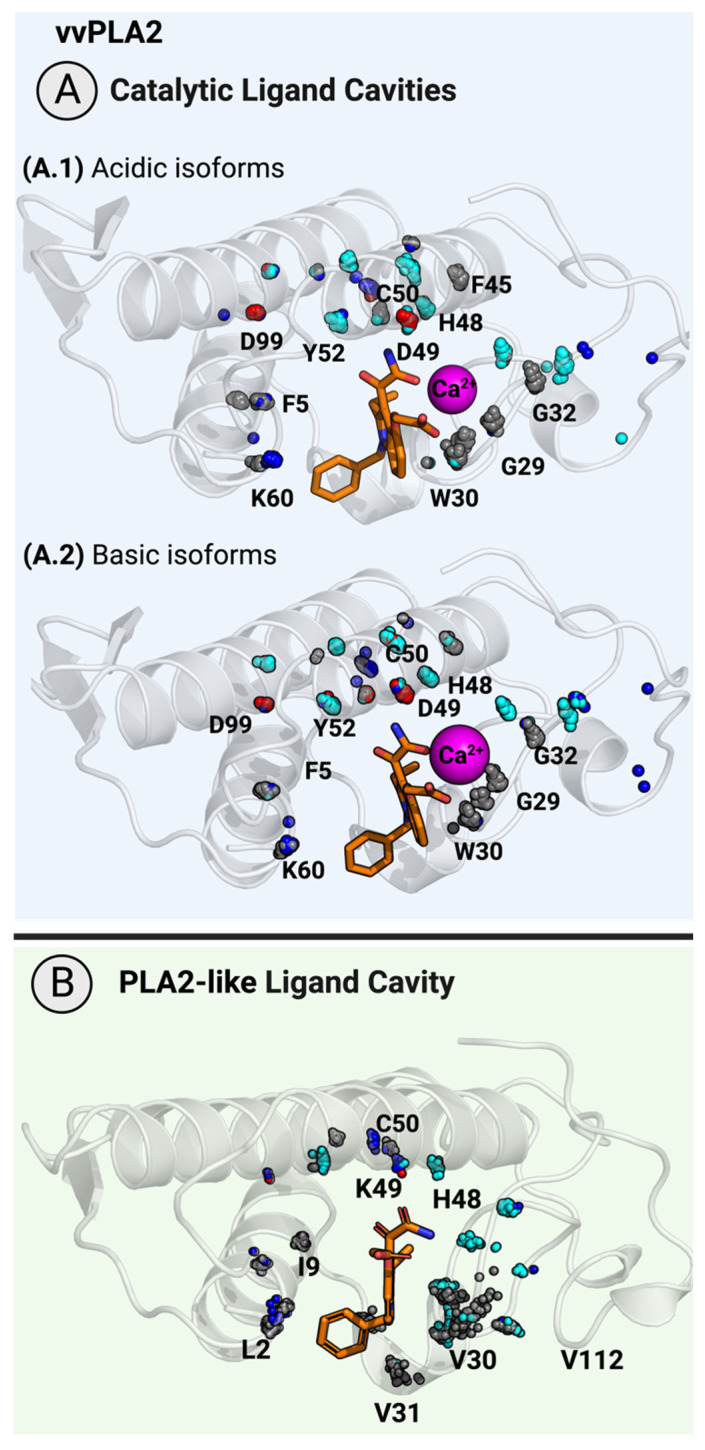
(**A**) Catalytic and ligand-binding cavity in vvPLA2 and (**B**) PLA2-like proteins. The protein is represented as a cartoon, and varespladib, a broad-spectrum sPLA2 inhibitor, is shown as orange sticks. The residues located within a radius of 7 Å of the catalytic cavity are shown as spheres. The sphere position represents the atomic coordinates of the Cα atom. The color scheme and numbering are the same as in Figure 6. The figure clearly shows that residues of the same nature are present in the same location in most protein databases, as can be seen by the frequent aggregation of spheres of the same color.

Understanding the properties of a ligand cavity guides the rational design of specific inhibitors. Ideally, the inhibitor should be effective against the vvPLA2 enzymes of a broad range of species (or, at least, for a wide range of species in each geographic region). Therefore, conserved areas of the binding pocket must be explored. The analysis of the ligand cavity showed a similar shape and volume for all the vvPLA2 and PLA2-like proteins included in this study. Moreover, the residues that delimit the cavity are highly conserved (Figure 7). For the acidic vvPLA2, we highlight the importance of residues 2, 5 (N-terminus), 26–32 (Ca^2+^ loop), 44–45 (a pair of consecutive cysteines), 47–51 (which include the catalytic triad and the invariant adjacent residues: Val47 and Cys51), 59 (Asp 45%, Asn 40% or Ser 15%), and 60 (Pro 95%) and the Ca^2+^ ion. These residues delimitate the cavity in more than 90% of the sequences analyzed. To a lesser extent, we identified the invariant residues Tyr52 and Asp99 (Figure 7A). Regarding the basic vvPLA2 enzymes, we found relevant interactions with Leu2, Ile5, the Ca^2+^ loop (26–32), Cys44, Cys45, Val47, His48, Asp49, Cys51, Asn59 (50%) or Ser59 (20%), Pro60 (40%) or Thr60 (40%), Lys61 (95%), Asp99 (100%), and the Ca^2+^ ion, conserved in >80% of the models inspected (Figure 7B). Therefore, almost all the conserved residues of the binding cavity are common to acidic and basic isoforms.

In the case of the PLA2-like proteins, once again the interactions are restricted to a smaller zone, which comprises residues Leu2 or Val2, Leu5 (and to less extent Phe5), Gly6 (60%), Ile9 (at the N-terminus), Pro17 (65%) or Ala17 (35%), a hydrophobic residue at position 18, Thr19 or Lys19 (~35% equal probability), Ser20 (76%), the invariant Tyr21, Gly22 (97%), Cys23 (100%), 27–30 (equivalent to the Ca^2+^ loop), Cys44 (100%), His48, Lys49 (Ser or Gln), Thr112 (100%), Lys116 or Arg116 (which together show conservation >85%), and, to a lesser extent (74%), the residue Tyr52.

The extent of conservation of the interactions in the binding cavity can be even better perceived in Figure 7, where spheres of the same color aggregate together, meaning that residues of the same nature are essentially found in similar spatial locations.

In the two X-ray structures of PLA2-like proteins co-crystalized with varespladib (6PWH [74] and 7LYE [76]), the ligands form hydrophobic contacts with residues Leu2, Leu5, Ile9, and Leu96 and H-bonds with Asn27 (backbone), His47 (ND1), and Lys48 (at NZ). In line with our results described above, all these residues are within the most strictly conserved core (>85). Thus, we can justify, at least in part, why varespladib, the only small drug-like PLA2 inhibitor under clinical trials for treating snakebite envenoming, possesses such a broad interspecific activity, inhibiting the PLA2s of 28 medically important snakes from six continents [76]. In sum, the analysis of all the structures of this database supports the possibility of finding broad-spectrum drug-like vvPLA2 competitive inhibitors, because most interacting sites at the substrate-binding pocket are sequentially and spatially preserved to a great extent (Figure 7), and the interaction of varespladib with the PLA2s validates that conclusion. Furthermore, a comparative analysis of the binding cavity along the three distinct vPLA2 isoforms reveals that residues appearing frequently in the analysis are highly conserved, as is the case of Leu2, Phe5, the glycines and cysteines of the calcium loop and equivalent region in the PLA2-like proteins, His47, and Lys60.

### 2.5. The N-Terminal Region

**Figure 8:**
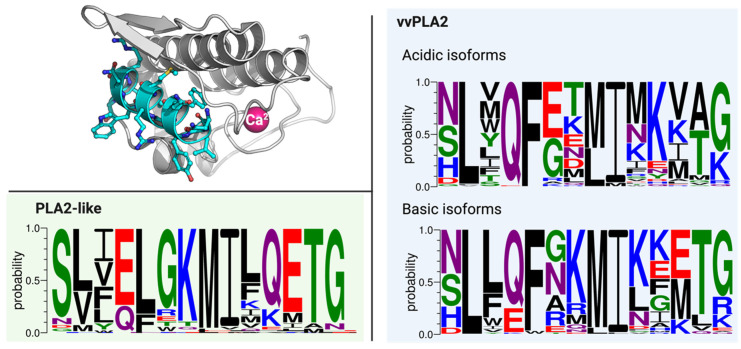
(**Top Left**) A vvPLA2 enzyme in grey cartoon highlighting the N-terminal region (in blue). (**Right**) The sequence for the same segment in vvPLA2s. (**Left Bottom**) The sequence for the PLA2-like proteins.

The overall conservation of the N-terminal region of the sequence is small, even though some of its residues are remarkably conserved. In acidic and basic vvPLA2s, there are only three highly conserved residues across the database: Leu2, Phe5, and Ile9. These residues are located at the hydrophobic tunnel of vvPLA2 that leads to the active site. The residue at position 4 is almost always Gln in the acidic vvPLA2s and is changed for a Glu only in 20% of the basic vvPLA2s. Residues at positions 6 and 7 are primarily negative and positive for acidic and basic vvPLA2s, respectively. The basic enzymes have more charged residues than their acidic counterparts. Finally, the two last residues are frequently either a threonine or a glycine (Figure 8).

More than 80% of the PLA2-like proteins display a serine at position 1, followed by a pair of hydrophobic residues.

Position 4 is occupied by a Glu (70%) or Gln (30%) residue. This ratio inverts on basic vvPLA2s. The invariant vvPLA2′s Phe5 is changed for a highly conserved Leu5 in PLA2-like proteins, followed by a very conserved motif of GKMI-X-QETG, where X is primarily a hydrophobic residue. The N-terminal region of the PLA2-like proteins is very much anionic and much more conserved than that in the vvPLA2 enzymes.

Site-directed mutagenesis of Phe5 and Ile9 in the bovine pancreatic PLA2 caused significant perturbations in the N-terminal conformation and the enzyme’s orientation toward the membrane. Furthermore, kinetic studies indicated that mutations at identical residues caused a substantial decrease in the rate of hydrolysis of micellar and vesicle substrates [53]. Moreover, the binding affinity of the mutant enzymes to the vesicle’s interface was not significantly affected; the perturbations in catalysis were manifested mainly in *k*_cat_ at the interface. This may justify why Phe5 is highly conserved in the vvPLA2s but replaced mainly by another residue (Leu5) in PLA2-like proteins, which does not need the residue to obtain a correct catalysis orientation.

### 2.6. The C-Terminal Region

**Figure 9:**
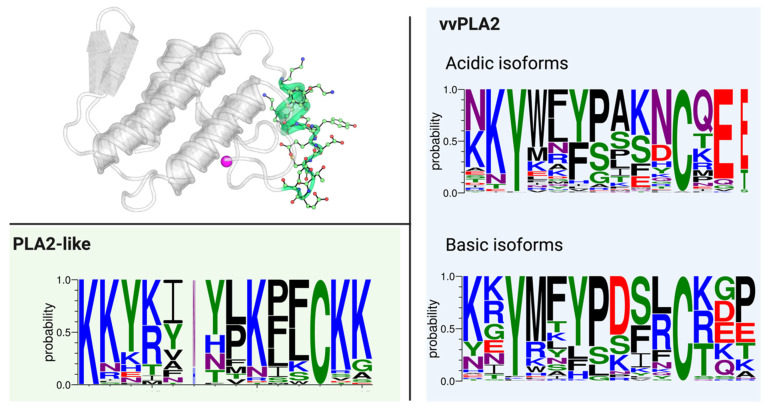
(**Top Left**) A vvPLA2 enzyme in grey cartoon with the C-terminus highlighted in green. (**Bottom Left**) The conservation of residues at the C-terminus of the PLA2-like proteins. (**Right**) The conservation for the same segment in vvPLA2s.

This C-terminal region (residues 115–129) is particularly relevant as it is the principal determinant for myotoxicity, the principal and most common pathophysiologic effect of PLA2-like proteins. In contrast, vvPLA2s’ myotoxicity is mostly driven by phospholipid hydrolysis. Therefore, a fundamental difference needs to exist in the C-terminal region of both categories of PLA2s. A close inspection of the database sequences clearly shows where the difference lies: the consistently large abundance of cationic residues and the inexistence of negative residues at the two tips of the C-terminal region of PLA2-like proteins in comparison with vvPLA2 enzymes (Figure 9). For more detailed information, please see Figures S6–S8.

Two positive residues at the beginning of the 115–129 region are found in 88% of the PLA2-like protein sequences, and two positive residues at the end of the 115–129 region are present in 77% of the same sequences. This contrasts strikingly with the same region of the vvPLA2 enzymes, in which such pairs of positive residues are found in 38% of the basic vvPLA2s and 27% in the acidic vvPLA2s. Further, negative residues are almost never found at the two end positions of the 115–129 region of PLA2-like proteins (3.5% of the sequences), in contrast with their presence in 46% of the basic vvPLA2s and 100% of the acidic vvPLA2s analyzed. In addition, the 115–129 region of PLA2-like proteins between the two positive tips is rich in hydrophobic/aromatic residues, interspersed with additional cationic residues.

**Figure 10:**
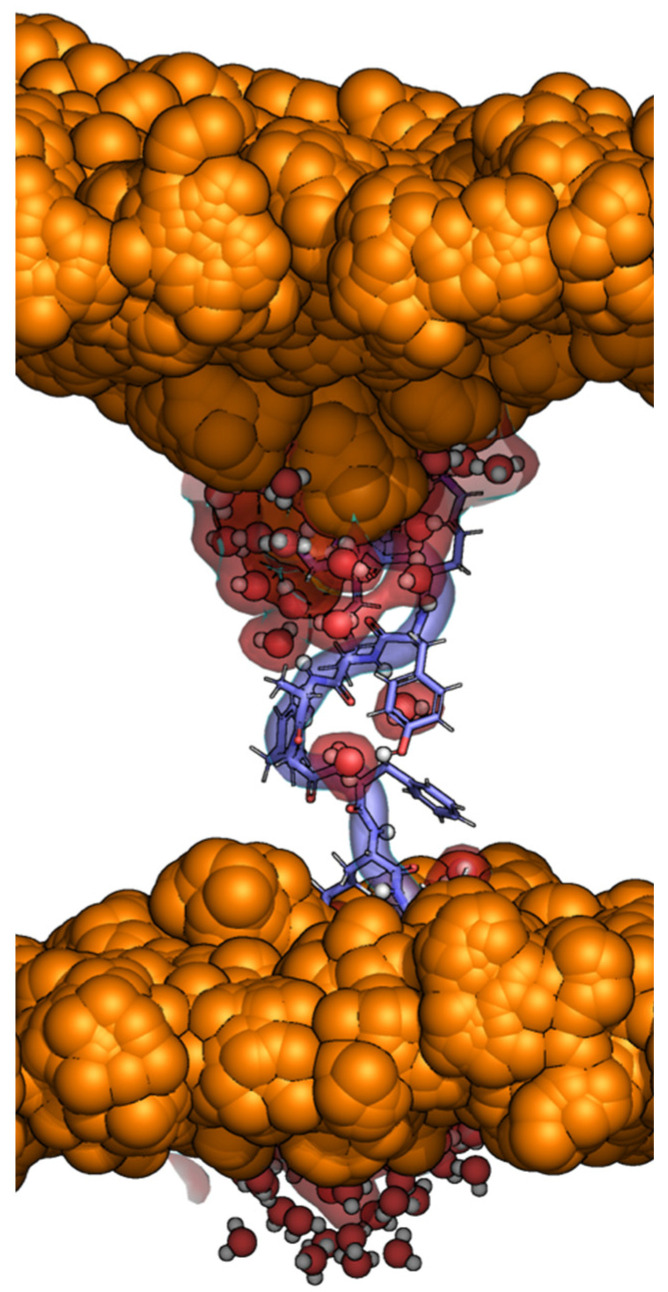
Molecular dynamics simulations of a positively charged (+6) peptide, shown in blue cartoon and sticks, which mimics the *C. oreganus abyssus* myotoxin II C-terminal segment inserted in a DOPS membrane (where the phosphorus atoms of multiple molecular dynamics configurations are represented as orange spheres). The peptide sequence KKYRIYPKFLCKK obeys the MT-II C-terminal pattern found for a large group of PLA2-like proteins, i.e., a pair of positive residues accompanied by one or two positively charged and hydrophobic aromatic residues, ending with another pair of positively charged residues. These characteristics may lead to membrane penetration and water permeation, as observed above, with the water molecules penetrating the membrane, evidenced by a red surface.

Earlier studies reported molecular dynamics simulations of a membrane-bound 13-residue peptide whose sequence mimics the C-terminus of the *C. oreganus abyssus* PLA2-like myotoxin II, and provided evidence of the likely importance of the sequence consensus found for the PLA2-like proteins concerning membrane disruption. The study results showed that the peptide fits very well in the thickness of a cell bilayer when inserted perpendicularly to the membrane plane. The positive tips engage in ionic hydrogen bonds with the phosphate groups of each cell bilayer face and with the hydrophobic region well across the hydrophobic bilayer core [99]. The simulations shed light on a potential permeabilization mechanism—as the peptide segment mimicking the 115–129 region is interspersed with aromatic and positive residues, the former may disturb the membrane packing, and the latter promote water leakage across the membrane due to their hydrophilicity (Figure 10).

Strikingly, the results obtained from the database analysis show that this sequence consensus is common for the C-terminal region of almost all the PLA2-like proteins. The results pictured in Figure 9 and Figure 10 indicate that this charge/hydrophobicity topology is shared among most of the sequences of the PLA2-like proteins in the database and is absent in the vvPLA2 enzymes. Consequently, this mechanism may be at the origin of the membrane-disrupting activity of the PLA2-like proteins, particularly if the C-terminal disulfide bridge is easily reduced or if the PLA2-like proteins penetrate the membrane deeply enough to bury the C-terminal segment extensively.

This hypothesis is further strengthened by the known lack of activity of PLA2-like proteins towards zwitterionic vesicles and high activity towards negative vesicles; i.e., the 115–129 positive tips anchor well to the heads of negative vesicles but have unfavorable interactions with the positive tips of zwitterionic vesicles. In further agreement, the abundant negative residues (Asp/Glu) found at the two ends of the C-terminus of vvPLA2 enzymes might interact with the membrane phosphates if the C-terminus of vvPLA2s transverses the membrane. Such electrostatic repulsions hinder the penetration of the vvPLA2s’ C-terminus. Nevertheless, despite all the evidence, equating the toxicity of the small peptides to the whole protein is not straightforward; further research is needed to clarify definitely the mode of action of PLA2-like proteins.

In earlier works of Fontes and coworkers, it was proposed that the PLA2-like proteins use a cationic membrane-docking site and hydrophobic membrane-disruption site to permeabilize the membrane [36,37,63,65]. This mechanism assumes the importance of a protein dimer at the membrane interface and a conformational transition upon ligand binding. Nevertheless, even though dimerization and conformational transitions may be relevant for PLA2-like myotoxicity, particularly in the complex in vivo environment, the fact that short peptides can recapitulate the full enzyme activity often suggests that the specific properties of the 115–129 sequence are at the core of the myotoxic activity of the PLA2-like proteins.

In a detailed analysis of individual sequences (Figure S8), we see that 93% of the PLA2-like proteins have the distinctive characteristics we propose as necessary to penetrate and destabilize the membrane:

i. Positive charge at each tip;
ii. Absence of negative charge at the tip;
iii. Positive and aromatic residues interspersed at the C-terminus core.

These same conditions are only met in 9% of the vvPLA2 enzymes. Within these 9% of vvPLA2s, eight of them stand out for having two positive charges at each tip: vvPLA2s from seven *Bothrops* vipers *(B. asper*, *B. brazili*, *B. jararacussu*, *B. moojeni*, two isoforms of vvPLA2s from *B. marajoenis, B.pirajai*) and *Bothrocophias hyoprora*. These properties seem to be ideal for membrane disruption and are shared with 36% of the PLA2-like proteins. It would be interesting to investigate if the latter vvPLA2s induce myotoxicity through hydrolytic and non-hydrolytic mechanisms, as they possess molecular determinants for both modes of action. In this scenario, it is tempting to speculate that such proteins are an evolutionary link between the ancestral myotoxicity mechanism (phospholipid hydrolysis) and the more modern and viper-exclusive mechanism of toxicity, non-hydrolytic membrane destabilization.

## 3. Conclusions

We have used homology modeling and molecular modeling to create a database of the tridimensional structure of 217 vvPLA2 enzymes and PLA2-like proteins. The structures developed here go beyond the typical X-ray structures found in the Protein Data Bank because they all include the crucial Ca^2+^ cofactor and have the Ca^2+^ loop modeled in the active conformation; above all, the proteins were modeled in the relevant physiologic environments, both in solution and bound to the cell membrane. Therefore, they provide information that goes much beyond what can be found in the Protein Data Bank. The database is freely available for download, to the scientific community, providing a resource for further studies within the community.

An initial database analysis of the protein sequences and structures performed here identified many essential aspects of the anatomy of these central toxins, such as the conservation of the interactions at the binding site, which may provide critical clues for the development of a universal interspecific inhibitor against PLA2 snakebite envenoming, or clear indications about the specific motifs behind the myotoxic activity of PLA2-like proteins. The database and its analysis already revealed the diversity and conservation among PLA2 toxins, guiding the discovery of small-molecule inhibitors targeting their diverse mechanisms of action. It may reveal many more well-hidden secrets about the remarkable versatility of the PLA2 protein family.

## 4. Methods

### 4.1. Selection of the Protein Sequences

We started the vvPLA2 enzyme and PLA2-like protein database assembly by searching sequences in the UniProt database [81,82] (up to May 2023), using the keywords “Phospholipase A2” and “Viperidae”. Only the “Reviewed” sequences were selected, totaling 346 sequences from 79 species. We split the sequences into three categories:

i. Complete sequences of vvPLA2 enzymes;
ii. Complete sequences of PLA2-like proteins;
iii. Incomplete sequences (fragments) of either vvPLA2 enzymes or PLA2-like proteins.

Only the mature chains were used, and the signal peptide was deleted. We further divided the first group (complete vvPLA2s) into two subgroups with acidic or basic isoforms. The sequences of categories (i) and (ii) were used in this study and are provided in an editable Supplementary Materials.

### 4.2. Benchmarking the Homology Modeling Protocol

The design of a robust and accurate pipeline for the generation of the 3D structures for the protein sequences played a crucial role. We started by determining the sequence identity threshold of the homology modeling templates needed to obtain accurate 3D protein models. Accordingly, we selected 6 vvPLA2s and 6 PLA2-like proteins with known 3D structures from distinct viper species (25% of the non-redundant 3D structures deposited in the PDB until May 2023 and >10% of the sequenced species). Then, we checked how well these structures could be predicted by comparing the predictions with the known 3D structures as a function of the sequence identity of the templates. We also determined the sequence similarity threshold to obtain high-accuracy homology modeling predictions, and we established that templates with a sequence identity >55% resulted in precise models. The benchmarking details are given in Tables S2–S4.

### 4.3. The Pipeline for Building the 3D Structures of the Database Proteins

**Figure 11:**
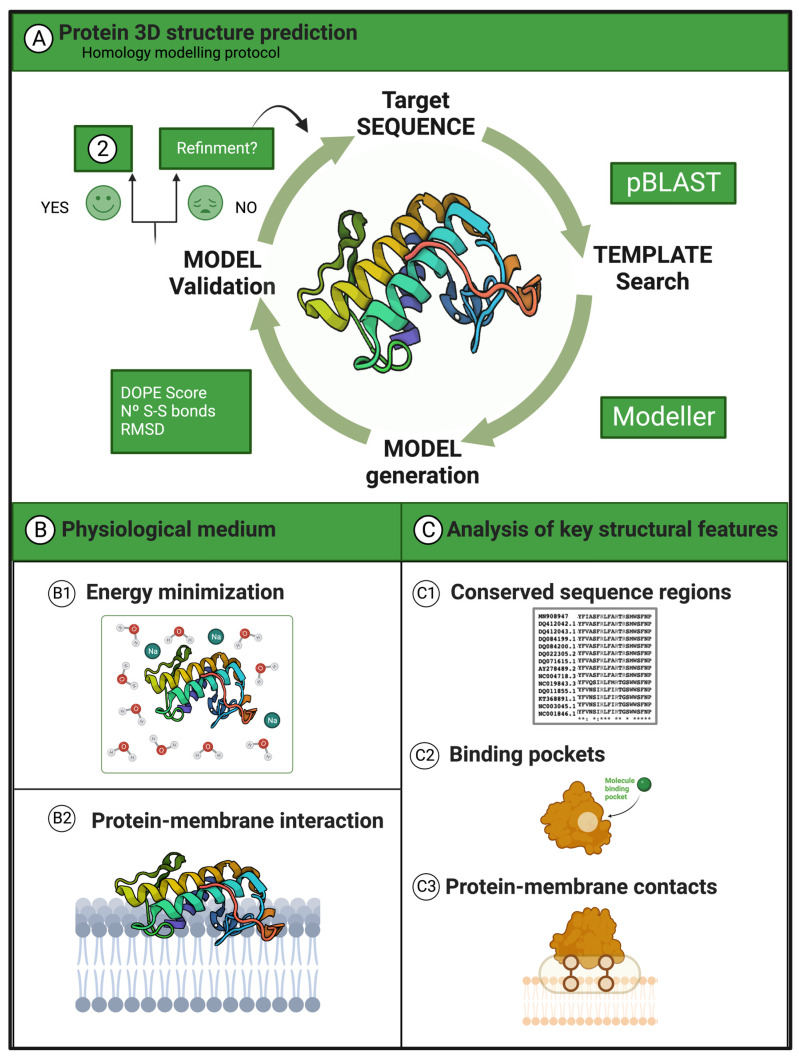
Workflow for generating the initial models and subsequent analyses. (**A**) Summary of the protocol for predicting the protein 3D structures. (**B**) Solvation of the models in a water box and energy minimization (three cycles) using the Amber18 software. After the minimization, we predicted the binding position of the enzyme in a phospholipid bilayer membrane. (**C**) Analysis of important sequence and structural motifs in the proteins.

We designed a semi-automatic pipeline with bash-shell and biopython [100] scripts to search for templates and generate the protein structures using modeller9.25 [101,102]. Ultimately, we carried out a final visual inspection using PyMol 2.5 software [103]. Figure 11 illustrates the protocol.

#### 4.3.1. Obtaining Modeling Templates for vvPLA2 enzymes and PLA2-like Proteins

The templates were selected based on the results of the benchmarking protocol. In sum, for each UniProt entry, and considering only the sequence region that encodes the mature protein, we searched for similar proteins with a known 3D structure available on the PDB database [104] using the blast tool [105] and the biopython [100] modules SeqIO and SearchIO. In general, the sequence coverage was near 100%. First, the template entries with higher sequence identity were selected and classified into the High-Quality (sequence id. > 70%), Good-Quality (sequence id. = 55–70%), or Medium-Quality class (sequence id. < 55%). Then, we selected a maximum of 5 templates of the highest quality group found for each protein target. This criterion was implemented to avoid templates with lower-quality groups compromising the final model’s accuracy. As described in the Results section, using High-Quality or Good-Quality templates generates 3D homology models with an accuracy comparable to good-resolution X-ray structures.

#### 4.3.2. Alignment of the Target and Templates

The alignment of the templates with the target proteins was performed using Modeler 9.25 software [101,102]. For the vvPLA2 enzymes, we generated the alignments by building a chimera-like template protein because most of their X-ray structures lack the Ca^2+^ cofactor and have the Ca^2+^ loop (residues 23–40) in an open conformation. Hence, residues 23–40 were replaced by their counterparts in the X-ray structures of the vvPLA2s that have the Ca^2+^ cofactor co-crystallized (PDB codes: 1OZ6 [31], 1BK9 [106], 1PSJ [107,108], and 1JIA [108]).

#### 4.3.3. Creating the Homology Models and Optimizing Their Structure with Molecular Mechanics

In the end, five models of each sequence were built. Those with the correct number of disulfide bonds and the best DOPE scores [109] were chosen and immersed in an octahedral box of TIP3P water molecules [110], with a solvation radius of 15 Å beyond any protein atom, using the tleap module of the Amber software package [111] and the Amber ff14SB force field [112]. Subsequently, energy minimization calculations produced models with relaxed geometries that were obtained in three steps in which hydrogen atoms, water molecules, and, finally, the whole system were minimized. A file with details on the 217 modeled, solvated, and minimized vvPLA2 and PLA2-like structures, specifying the species, genus, used templates, structural details, isoelectric point, overall sequence alignment, and atomic coordinates in PDB format, is available as Supporting Information.

### 4.4. Analyses of 3D Structures

#### 4.4.1. Topology

We visually inspected the global fold of the vvPLA2 and PLA2-like proteins, which is, on the whole, constituted by three alpha-helices and a beta-wing region, and created a matrix of root mean square deviation (RMSD) values for the atomic positions using Pymol software [103]. We also checked if the models correctly formed seven disulfide bridges (six for a few exceptions from the *Bitis* and *Trimeresurus* genera).

#### 4.4.2. Catalytic Pocket and Ligand Cavities

The vvPLA2 enzymes’ catalytic pocket residues were defined as those within a 7 Å radius of the Ca^2+^ ion. All residues with at least one atom within the cutoff were included. The residues were classified into five groups: hydrophobic (Ala, Gly, Leu, Ile, Met, and Val), aromatic (Phe, Tyr, and Trp), polar (Asp, Glu, Pro, Ser, Thr, and Cys), positive (Arg, His, and Lys), and negative (Asp and Glu). We defined a point in the hydrophobic channel, 3 Å away from the Ca^2+^ cofactor, to search for a ligand cavity. 

The evaluation of a ligand-binding cavity in the PLA2-like proteins comprised the same region as in the active forms.

#### 4.4.3. C-Terminal Segment

To calculate the sequence identity of the C-terminal region and to understand the sequence and structural features behind its membrane-damaging activity, we carried out a sequence alignment and comparative sequence analysis of the region comprising residues 115 to 129 (standard PLA2 numbering), and we quantified the conservation of residues for vvPLA2 and PLA2-like proteins.

#### 4.4.4. Protein–Membrane Interface

To determine the residues comprising the protein–membrane binding interface, we selected the *Echis carinatus* (PDB ID: 1OZ6 [31]) vvPLA2 enzyme as the lead structure and modeled a solvated vvPLA2–membrane (POPS) complex using the Charmm-Gui webserver [113] (details in Figure S4). Therefore, we uploaded the X-ray structure and estimated the positioning of the protein in the membrane using the PPM server [94,113,114,115]. This software calculates the orientation of the protein on the membrane based on known 3D structure complexes and their sequence similarity. We then solvated the system with a layer of 25 Å of TIP3P water and neutralized the system with CaCl_2_ at a concentration of 0.15 M, using the distance placement method. The final size of the system was 90.15 × 90.15 × 108.03 Å. We modeled the protein–membrane complexes for all vvPLA2 enzymes in the database by superimposing each protein on the *E. carinatus* vvPLA2–membrane complex and minimizing the energy of the protein–membrane complexes. Finally, we modeled the protein–membrane complexes for the PLA2-like proteins in the same way as described for the vvPLA2 enzymes, using as the lead structure the *B. asper* myotoxin II (PDB ID: 1Y4L [57]). All remaining conditions were similar. The final size of the latter system was 90.31 × 90.31 × 110.9 Å.

## Figures and Tables

**Table 1:** Comparison between the models of 21 PLA2 monomers taken from 12 different vvPLA2 and PLA2-like proteins and the corresponding X-ray structures. Models from Medium-Quality templates were created for all proteins, Good-Quality templates for 14 proteins, and High-Quality templates for 7 proteins. The difference in the number of modeled structures reflects, in some cases, the lack of Good- and High-Quality templates. The individual results are given in Tables S2 and S3.

Template Quality	<RMSD (Å)>	RMSD_MAX_ (Å)	No. Sequences	Correct No. SS Bridges
Medium-Quality (<55%)	1.6 ± 0.7	2.8	21	12 out of 21
Good-Quality (55–70%)	1.0 ± 0.2	1.3	14	14 out of 14
High-Quality (>70%)	0.8 ± 0.3	1.1	7	7 out of 7

## Data Availability

Data is contained within the Supplementary Materials.

## Supplementary Materials

The following supporting information can be downloaded at: https://www.mdpi.com/article/10.3390/toxins16020071/s1, Figure S1: Two possible dimerization interfaces of vvPLA2 enzymes; Figure S2: The three principal regions of the PLA2-like proteins; Figure S3: Phospholipase A2 bound to transition-state analogs; Figure S4: Protein–membrane system; Figure S5: Modeling of the PLA2-like protein from *B. pauloensis*, which adopts an extended dimer conformation, bound to the membrane; Figure S6: C-terminal sequence analysis of the vvPLA2s’ acidic subgroup; Figure S7: C-terminal sequence analysis of the vvPLA2s’ basic subgroup; Figure S8: C-Terminal sequence analysis of the PLA2-like proteins’ subgroup. Table S1: Surface area of seven dimeric vvPLA2 and PLA2-like proteins and their interface area; Table S2: Validation of the molecular modeling protocol for vvPLA2 enzymes as a function of the template quality; Table S3: Validation of the molecular modeling protocol for PLA2-like proteins as a function of the template quality; Table S4: Comparison between the homology modeling and the AlphaFold2 models. Table S5: All homology models vs. AlphaFold2 structures; Table S6: i-face qualification and quantification contacts. References [116,117,118,119,120,121,122,123,124,125,126,127] are cited in the Supplementary Materials.
