# Correction: de Oliveira et al. Viper Venom Phospholipase A2 Database: The Structural and Functional Anatomy of a Primary Toxin in Envenomation. *Toxins* 2024, *16*, 71

**DOI:** 10.3390/toxins16060250

**Published:** 2024-05-28

**Authors:** Ana L. Novo de Oliveira, Miguel T. Lacerda, Maria J. Ramos, Pedro A. Fernandes

**Affiliations:** Requimte-Faculty of Sciences, University of Porto, Rua do Campo Alegre s/n, 4169-000 Porto, Portugal; anoliveira@fc.up.pt (A.L.N.d.O.); up201605225@g.uporto.pt (M.T.L.); mjramos@fc.up.pt (M.J.R.)

## Additional Supplementary Materials

In the published publication [1], the PLA2 viper venom database does not include the submitted 3D structures of the proteins in the Supplementary Materials due to a mistake in the publishing process. The corrected Supplementary Materials appears below. The authors state that the scientific conclusions are unaffected. This correction was approved by the Academic Editor. The original publication has also been updated.

**Supplementary Materials:** The following supporting information can be downloaded at: https://www.mdpi.com/article/10.3390/toxins16020071/s1, Figure S1: Two possible dimerization interfaces of vvPLA2 enzymes; Figure S2: The three principal regions of the PLA2-like proteins; Figure S3: Phospholipase A2 bound to transition-state analogs; Figure S4: Protein–membrane system; Figure S5: Modeling of the PLA2-like protein from *B. pauloensis*, which adopts an extended dimer conformation, bound to the membrane; Figure S6: C-terminal sequence analysis of the vvPLA2s’ acidic subgroup; Figure S7: C-terminal sequence analysis of the vvPLA2s’ basic subgroup; Figure S8: C-Terminal sequence analysis of the PLA2-like proteins’ subgroup. Table S1: Surface area of seven dimeric vvPLA2 and PLA2-like proteins and their interface area; Table S2: Validation of the molecular modeling protocol for vvPLA2 enzymes as a function of the template quality; Table S3: Validation of the molecular modeling protocol for PLA2-like proteins as a function of the template quality; Table S4: Comparison between the homology modeling and the AlphaFold2 models. Table S5: All homology models vs. AlphaFold2 structures; Table S6: i-face qualification and quantification contacts. File S1: 3D-PDB structures of the 217 vvPLA2 and PLA2-like proteins analyzed in the database. The PDB file is named according to the UNIPROT code of the corresponding structure. References [116–127] are cited in the Supplementary Materials.
