# Supplementary material for: Viper Venom Phospholipase A2 Database: The Structural and Functional Anatomy of a Primary Toxin in Envenomation
Source: Toxins (Basel). 2024 Feb 1;16(2):71. doi: 10.3390/toxins16020071 (PMC10893444; doi:10.3390/toxins16020071)
Supplement: Supplementary file 1 [file toxins-16-00071-s001.zip › toxins-2657947-supplementary.pdf]

Supplementary Materials: Viper Venom Phospholipase A2 Database: The Structural and Functional Anatomy of a Primary Toxin in Envenomation

Ana L. Novo de Oliveira, Miguel T. Lacerda, Maria J. Ramos and Pedro A. Fernandes

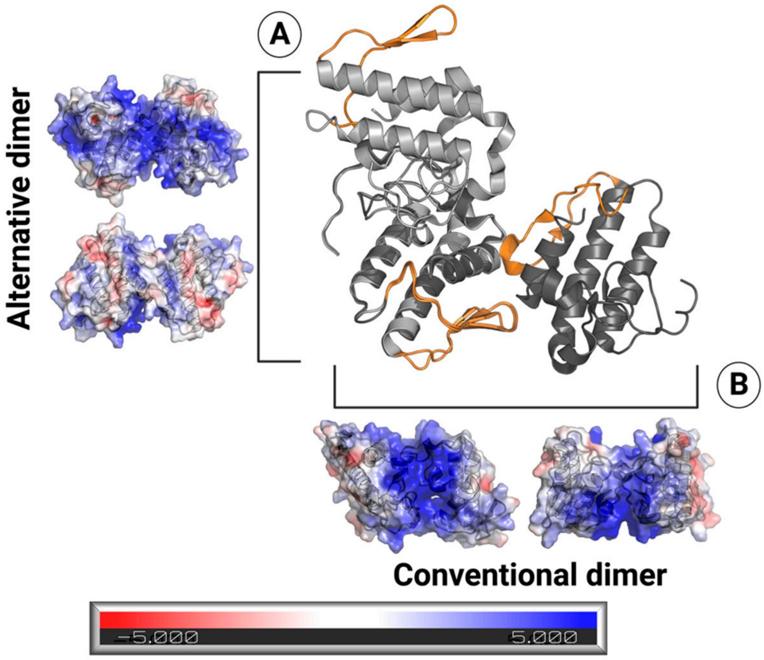

Figure S1. Two possible dimerization interfaces of vvPLA2: **(A)** The compact dimer. **(B)** The extended dimer. The right and bottom parts of the figure show the electrostatic potential projected over the molecular surface of the dimer from two different perspectives (bar units: k_BT/e^{-1}). The electrostatic surface was calculated with the APBS electrostatics plugin for Pymol 2.0, using default parameters[1]. The figure shows clearly that the positively charged residues are predominant on one side of the dimer in the case of the compact dimer conformation, compatible with the membrane association, and buried in the dimer interface in the extended dimer. Lastly, the electrostatic attraction with the negative phospholipid residues would only occur in a small region.

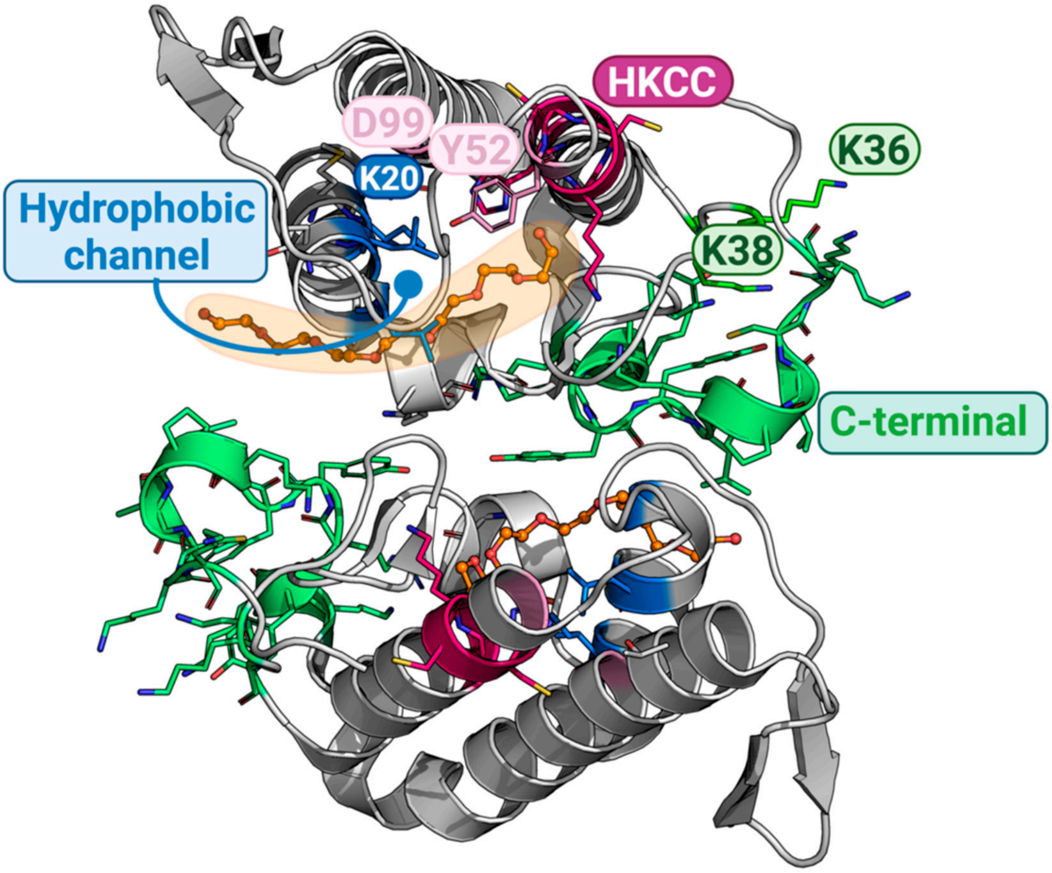

Figure S2. The three principal regions of the PLA2-like proteins. The figure shows the structure of the PDB—ID 1Y4L[8], which was solved in the compact dimerization conformation. The N-terminal and the three principal residues (in sticks) that define the hydrophobic channel are shown in blue. The HKCC motif is represented in magenta, and the residues Y52 and D99 stabilize the H48 residues through an H-bond network. The K36 and K38 residues and the C-terminal segment 115-129 are the main ones responsible for the PLA2-like myotoxicity and are highlighted in green. Finally, in orange, we show an organic molecule that mimics the region that the phospholipid should occupy.

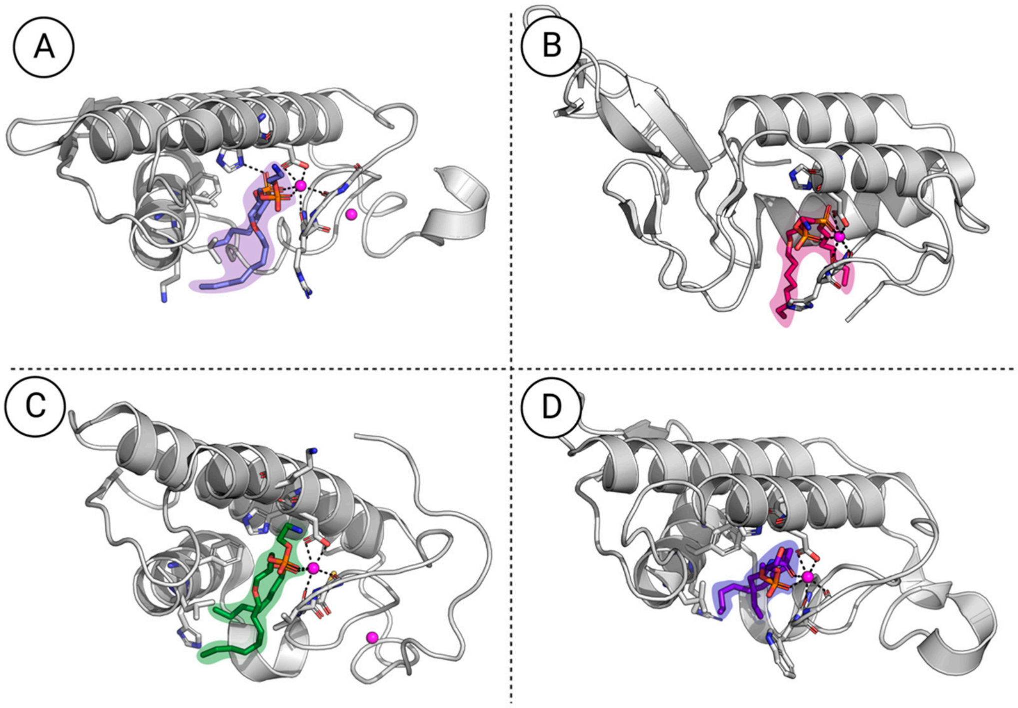

Figure S3. Phospholipase A2 bound to the transition-state analogs. The enzyme's secondary structure is represented in cartoon, and the residues that delimitate the ligands are shown as grey sticks. **(A)** The Indian cobra (*Naja naja*, PDB ID. 1POB[10]**); (B)** Human (PDB ID. 1POC[11])**, (C)** Bee (*Apis mellifera,*[12] PDB ID. 1POE); and **(D)** The wild-boar (*Sus scrofa)* PLA2 bound to substrate-derived inhibitor (PDB ID. 5P2P[13]).

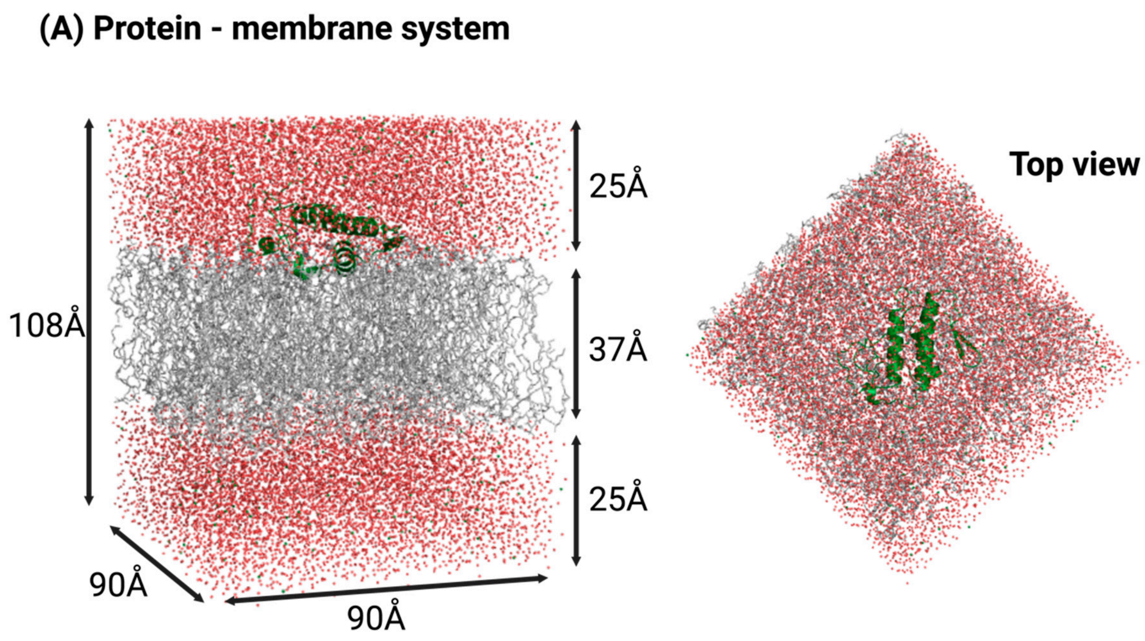

Figure S4. An acidic vvPLA2 from *E. carinatus* (PDB ID. 1OZ6, shown in the figure) and a *B. asper* PLA2-like protein (PDB ID. 1Y4L) were inserted in a POPS bilayer using the Charmm-Gui webserver. The membrane size ensures enough space for protein mobility (90 Å x 90 Å). Finally, the full system was then solvated in a water box (TIP3P water model) with a thickness of 25 Å.

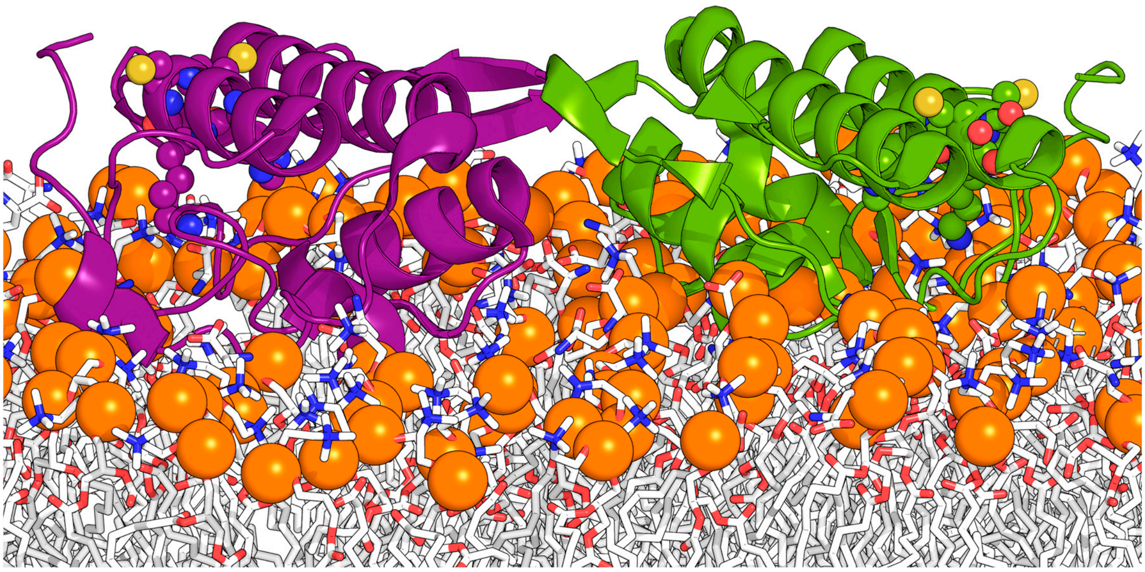

Figure S5. Modulation of the PLA2-like from *B. pauloensis*, which adopts an extended dimer conformation, bound to the membrane. (PDB ID. 1PA0[2]).

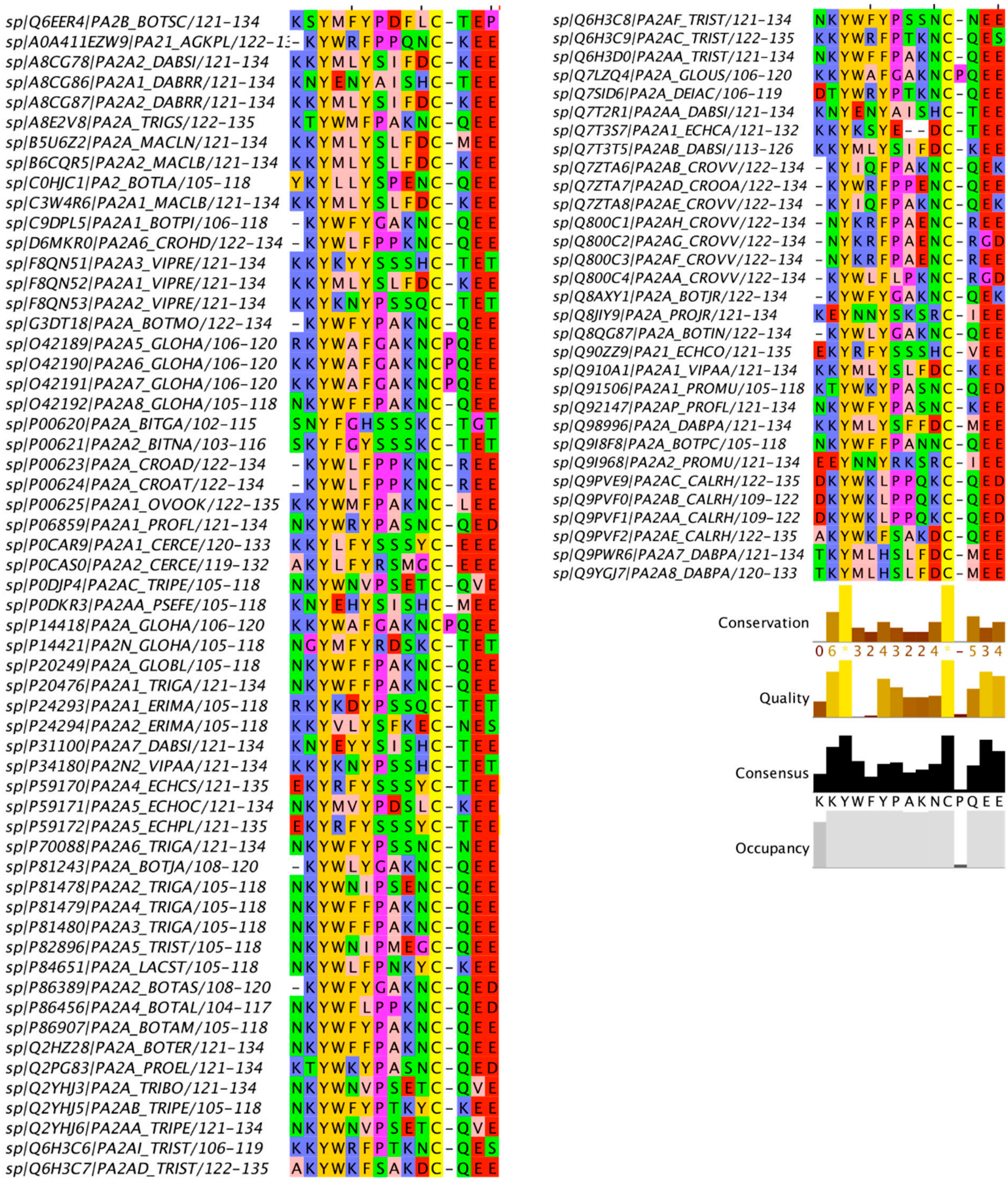

Figure S6. C-terminal sequence analysis of the vvPLA2 acidic subgroup. Aminoacid sequence of the C-terminal region (residues 115 to 129). The residues were colored according to their physicochemical properties using the Zappo default coloring range as implemented in JalView. Hydrophobic residues in salmon; aromatic residues in orange; positively and negatively charged residues in blue and red, respectively; hydrophilic in green; Pro and Gly in magenta; and cysteines in yellow. Each figure also indicates the consensus sequence (most frequent residues) and the residues occupancy (presence or absence of gaps).

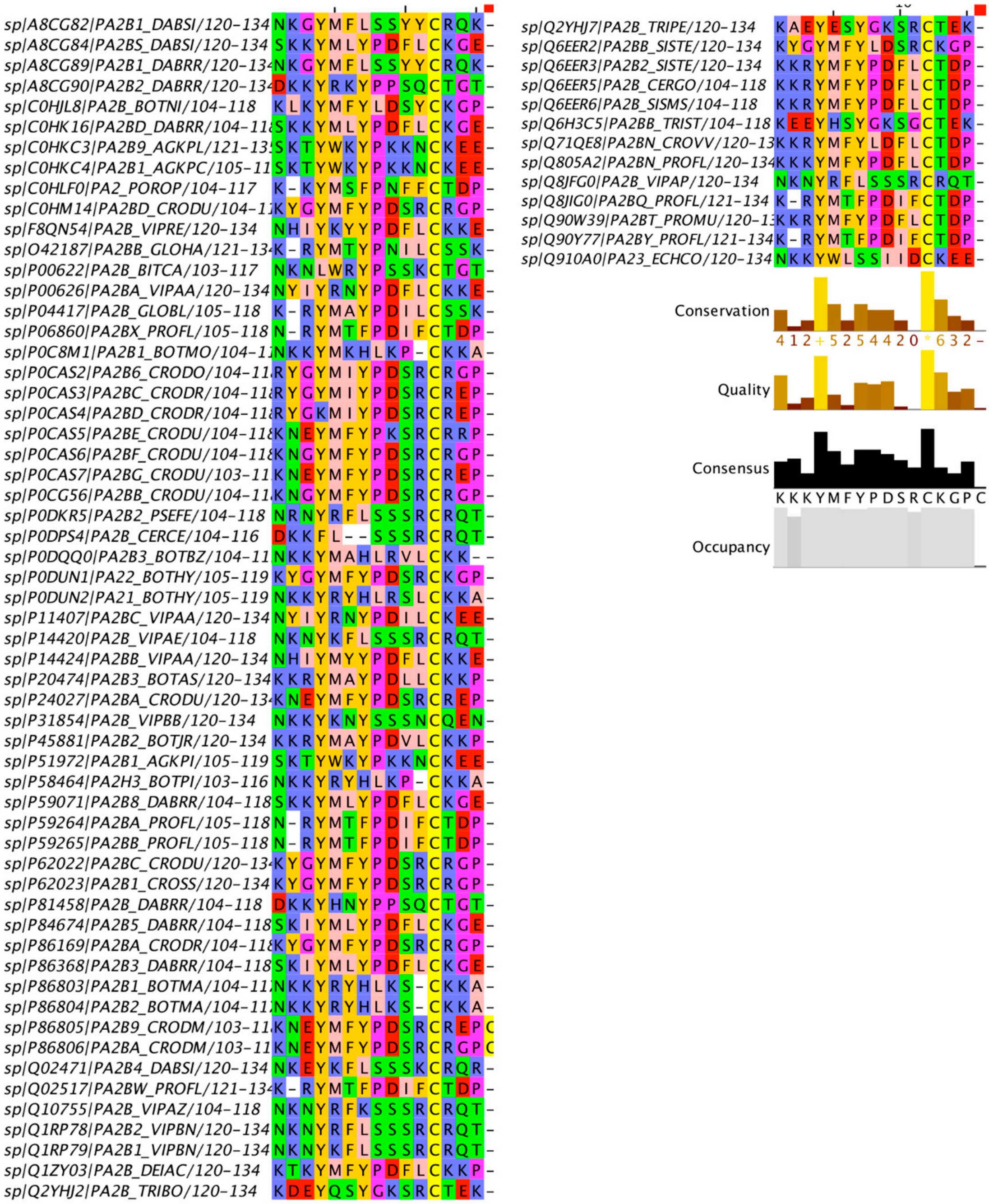

Figure S7. C-terminal sequence analysis of the vvPLA2 basic subgroup. Aminoacid sequence of the C-terminal region (residues 115 to 129). The residues were colored according to their physicochemical properties using the Zappo default coloring range as implemented in JalView. Hydrophobic residues in salmon; aromatic residues in orange; positively and negatively charged residues in blue and red, respectively; hydrophilic in green; Pro and Gly in magenta; and cysteines in yellow. Each figure also indicates the consensus sequence (most frequent residues) and the residues occupancy (presence or absence of gaps).

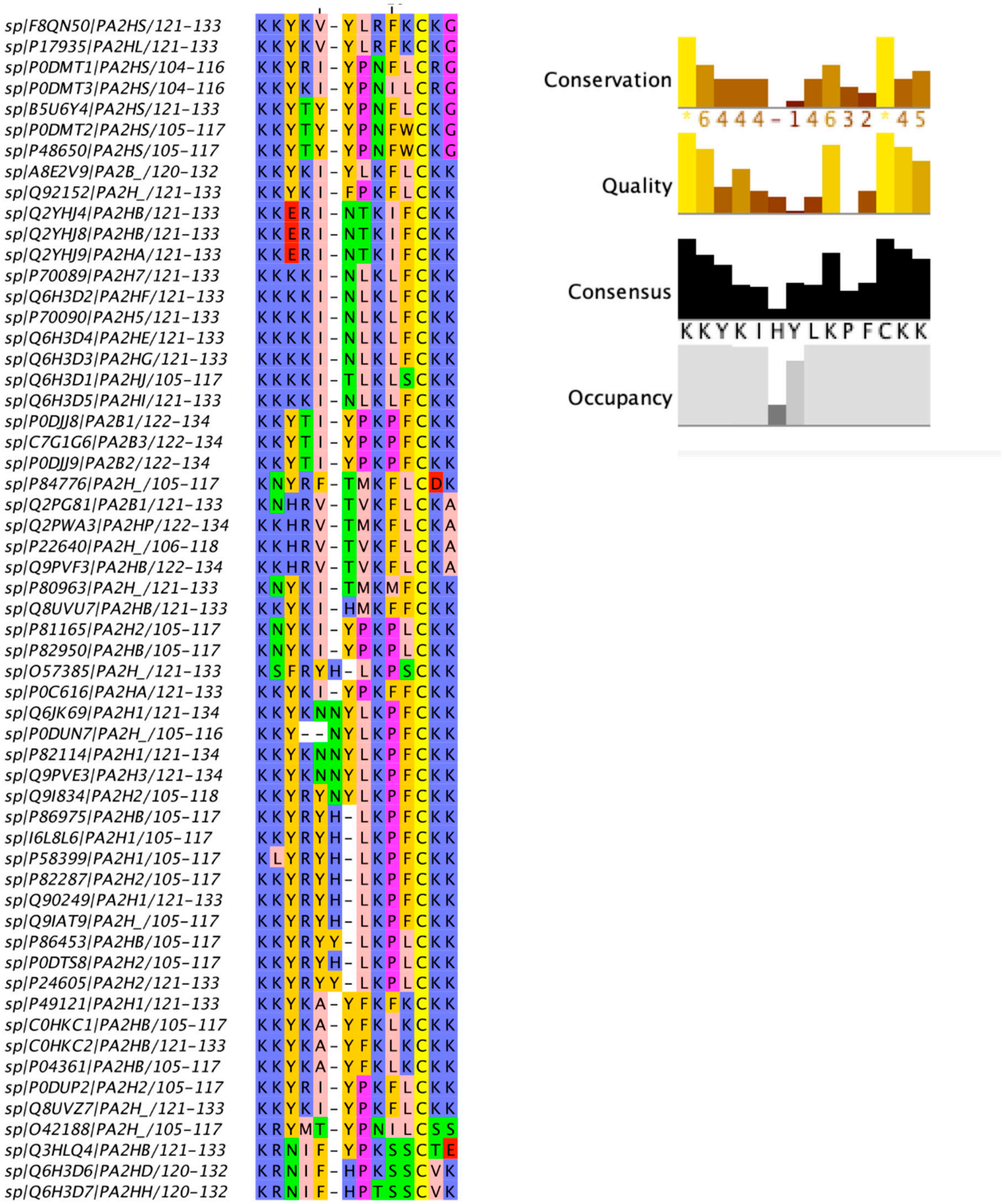

Figure S8. C-Terminal sequence analysis of the PLA2-like subgroup. Amino acid sequence of the C-terminal region (residues 115 to 129). The residues were colored according to their physicochemical properties using the Zappo default coloring range as implemented in JalView. Hydrophobic residues in salmon; aromatic residues in orange; positively and negatively charged residues in blue and red, respectively; hydrophilic in green; Pro and Gly in magenta; and cysteines in yellow. Each figure also indicates the consensus sequence (most frequent residues) and the residues occupancy (presence or absence of gaps).

Table S1. Surface area of seven dimeric vvPLA2 and PLA2-like proteins and their interface area (buried surface area upon dimerization), calculated as:.

$$Buried\ Area = (Area_{Chain\ A} + Area_{Chain\ B}) - Dimer\ Area_{Total}$$

The areas were computed using the GETAREA[1] webserver (https://curie.utmb.edu/getarea.html). The standard deviation is also shown. All the areas are given in Å^2.

sPLA2	Organism	X-ray structure	Dimer Form	Total Area	Polar Area	Apolar Area	Chain A Area	Polar Area	Apolar Area	Chain B Area	Polar Area	Apolar Area	Burried Area	AVERAGE	STD
PLA2-like	*B. pauloensis*	1PA0	Extended	12952	4621	8330	7061	2477	4584	6913	2493	4419	1022		
PLA2-like	*B. jararaccussu*	3JR8	Extended	12986	4520	8465	6787	2259	4528	7084	2602	4482	885	868	132
Basic vvPLA2	*A. halys pallas*	1JIA	Extended	13584	4510	9074	7087	2381	4705	7195	2433	4762	698		
PLA2-like	*B. pauloensis*	5VFH	Compact	12071	4392	7678	6847	2270	4576	6715	2246	4468	1491		
Basic vvPLA2	*B. asper*	5TFV	Compact	12140	4287	7853	6834	2315	4518	6664	2082	4582	1358		
Acidic vvPLA2	*B. jararaccussu*	1Z76	Compact	13929	5920	8008	7833	3124	4748	7676	2989	4686	1580	1393	163
PLA2-like	*B. asper*	1Y4L	Compact	12933	4877	8055	7050	2518	4531	7028	2526	4501	1145		
Pancreatic	Porcine	1FXF	Compact	12868	5245	7623	7156	2817	4338	7255	2895	4360	1543		

PDB IDs.: 1PAO[2], 3JR8[3], 1JIA[4], 5VFH[5], 5TFV[6], 1Z76[7], 1Y4L[8] and 1FXF[9].

Table S2. Validation of the molecular modeling protocol for vvPLA2 as a function of the template quality. To estimate the sequence identity threshold of the template necessary to obtain accurate 3D structures using our homology modeling method, we tried to reproduce known X-ray structures by applying templates with High-Quality (sequence id. > 70%), Good-Quality (sequence id. = 55-70%), or Medium-Quality classes (sequence id. < 55%). The software Modeler provides the DOPE score; The Interaction Score corresponds to the DOPE score divided by the number of heavy atoms. The Prosa II score from the ProsaWeb Server (https://prosa.services.came.sbg.ac.at/prosa.php; Nucleic Acids Research, 2007,Vol.35, WebServer issue W407–W410).

Species	Template Quality	Seq. length	Template Seq. ID	DOPE score	Interaction score	ProsaII score (Model)	ProsaII score (Template)	SS Bridges	RMSD backbone	PDB
Trimeresurus stejnegeri	medium	121	44	-1.12E+04	-2.47E-02	-5.61	-5.85	5	1.58	4RFP[14]
	good	121	55	-1.19E+04	-2.64E-02	-6.15	-5.29	7	1.11	
	good	121	69	-1.17E+04	-2.58E-02	-5.51	-5.50	7	0.98	
Vipera ammodytes meridionalis	medium	122	44	-1.09E+04	-2.40E-02	-4.86	-7.54	6	1.92	1JLT[15]
	medium	122	45	-1.13E+04	-2.44E-02	-4.75	-6.36	6	1.58	
	medium	122	53	-1.19E+04	-2.65E-02	-5.72	-4.91	7	1.17	
	good	122	55	-1.21E+04	-2.61E-02	-4.66	-4.13	7	1.17	
	good	122	65	-1.18E+04	-2.63E-02	-5.67	-4.13	7	1.04	
	good	122	68	-1.22E+04	-2.63E-02	-5.37	-5.47	7	0.93	
	high	122	93	-1.17E+04	-2.60E-02	-5.54	-5.29	7	0.55	
	high	122	92	-1.21E+04	-2.62E-02	-5.52	-5.03	7	0.34	
Daboia russelii	medium	121	43	-1.08E+04	-2.42E-02	-4.60	-5.85	6	2.07	1OXL[16]
	good	121	67	-1.20E+04	-2.70E-02	-4.76	-2.93	7	0.71	
	high	121	93	-1.17E+04	-2.64E-02	-4.97	-4.80	7	0.65	
Echis carinatus	medium	120	44	-1.04E+04	-2.31E-02	-4.85	-5.97	5	2.31	1OZ6[17]
	good	120	55	-1.15E+04	-2.55E-02	-4.75	-5.03	7	1.23	
	good	120	66	-1.15E+04	-2.54E-02	-4.88	-5.29	7	1.05	
Gloydius halys	medium	122	45	-1.03E+04	-2.22E-02	-4.99	-6.36	6	3.19	1B4W[17]
	good	122	55	-1.13E+04	-2.43E-02	-5.10	-4.13	7	1.14	
	good	122	69	-1.17E+04	-2.52E-02	-5.03	-4.87	7	0.52	
Daboia siamensis	medium	122	44	-1.10E+04	-2.47E-02	-4.72	-5.85	5	3.28	2H4C[18]
	medium	122	44	-1.17E+04	-2.46E-02	-5.02	-7.37	6	1.67	
	medium	122	54	-1.22E+04	-2.74E-02	-5.24	-4.91	7	1.04	
	medium	122	54	-1.25E+04	-2.63E-02	-5.48	-4.60	7	1.41	
	high	122	70	-1.23E+04	-2.76E-02	-5.24	-5.70	7	0.90	
	medium	122	69	-1.27E+04	-2.67E-02	-5.53	-5.03	7	1.13	
	high	122	89	-1.24E+04	-2.80E-02	-5.21	-5.29	7	1.03	

Table S3. Validation of the molecular modeling protocol for PLA2-like proteins as a function of the template quality. To estimate the sequence identity threshold of the template necessary to obtain accurate 3D structures using our homology modeling method, we tried to reproduce known X-ray structures by applying templates with High-Quality (sequence id. > 70%), Good-Quality (sequence id. = 55-70%), or Medium-Quality classes (sequence id. < 55%). The DOPE score and Interaction score were calculated as in Table S2.

Species	Chain	Template Quality	Seq. length	Template Seq. ID	DOPE score	Interaction Score	ProsaII score	ProsaII Template	SS bridges	RMSD backbone	PDB
Protobothrops flavorviridis	*A ,B ,C,D*	good	122	55	-1.17E+04	-2.57E-02	-5.5	-4.66	7	1.23	6AL3[19]
		high	122	64	-1.15E+04	-2.51E-02	-5.46	-5.14	7	1.27	
		high	122	80	-1.17E+04	-2.55E-02	-5.24	-5.2	7	1.04	
Bothrops moojeni	*A,B*	medium	122	54	-1.13E+04	-2.46E-02	-5.74	-4.78	7	1.51	1XXS[20]
		good	122	69	-1.12E+04	-2.45E-02	-5.31	-5.06	7	1.45	
		high	122	99	-1.17E+04	-2.55E-02	-5.46	-5.56	7	0.72	
	A	good	121	55	-1.17E+04	-2.57E-02	-5.77	-4.13	7	1.16	

Atropoides nummifer		good	121	67	-1.16E+04	-2.56E-02	-5.56	-2.93	7	1.06	2AOZ[21]
		high	121	91	-1.18E+04	-2.60E-02	-5.4	-5.63	7	0.74	
Cerrophidion godmani	*A*	good	121	55	-1.20E+04	-2.66E-02	-6.05	-6.41	7	0.97	1GOD[22]
		good	121	64	-1.17E+04	-2.59E-02	-5.70	-4.93	7	1.11	
		high	121	91	-1.20E+04	-2.66E-02	-5.6	-5.1	7	1.15	
Bothrops pirajai	*A,B*	medium	121	55	-1.12E+04	-2.46E-02	-6.04	-5.35	7	1.38	2Q2J[22]
		good	121	62	-1.10E+04	-2.41E-02	-5.91	-4.93	7	1.46	
		high	121	99	-1.14E+04	-2.49E-02	-5.45	-5.2	7	1.06	
Bothrops asper	*A,B*	medium	121	55	-1,14E+04	-2.51E-02	-5,83	-5,29	7	1.09	1Y4L[8]
		good	121	63	-1.12E+04	-2.47E-02	-5,82	-4,93	7	1.22	
		high	121	97	-1.17E+04	-2.58E-02	-5,45	-5,21	7	0.57	

Table S4. Comparation between the homology modelling models derived in this work and the AlphaFold[23] models. All the RMSD values are given in Å.

	**PDB**	**Ref.**	**RMSD between the models and the X-ray structures**	**RMSD between the AlphaFold and the X-ray structures**	**RMSD between the models and AlphaFold structures**
vvPLA2	1B4W	17	0.4	0.3	0.4
	1JLT_A	15	0.7	0.3	0.6
	1JLT_B	15	0.3	0.3	0.5
	2H4C_A	18	0.8	1.0	0.7
	2H4C_B	18	0.7	0.8	0.7
	1OXL	24	0.5	0.5	0.5
	1OZ6	16	0.5	0.4	0.5
	4RFP	14	0.6	0.7	0.6
PLA2-like	1XXS	20	0.4	0.6	0.4
	1GOD	25	0.8	0.7	0.4
	1Y4L	8	0.3	0.5	0.4
	2Q2J	22	0.3	0.4	0.4
	2AOZ	21	0.4	0.4	0.4
	6AL3	19	0.6	0.6	0.4

Table S5. All homology models vs AlphaFold2 structures. We have calculated the RMSD metrics between our modeled structures and the AlphaFold predicted models. Overall, both modeled structures present an RMSD lower than 1 Å, a value within the range of experimental errors associated with many X-ray structures. However, we found exceptions (highlighted in yellow);. A close inspection revealed that loop conformations are responsible for such high values.

**Acidic vvPLA2**		**Basic VVPLA2**		**PLA2-like Proteins**	
**UniProt Code**	**RMSD**	**UniProt Code**	**RMSD**	**UniProt Code**	**RMSD**
A8CG78	0.8	A8CG82	0.7	A0A1S5XW05	0.5
A8CG86	0.8	A8CG84	0.7	B5U6Y4	0.5
A8CG87	0.8	A8CG89	0.7	C0HKC1	0.4
A8E2V8	0.6	A8CG90	0.5	C0HKC2	0.5
B5U6Z2	0.8	C0HK16	0.7	F8QN50	0.6
B6CQR5	0.9	C0HKC3	0.6	O42188	0.3
C0HJC1	0.5	C0HKC4	0.6	P0C616	0.5
C3W4R6	0.7	C0HLF0	**2.2**	P0DKU1	0.5
C9DPL5	0.6	C7G1G6	1.7	P0DMT1	**4.5**
D0UGJ0	0.9	F8QN54	0.6	P0DMT2	0.5
D6MKR0	0.7	O42187	0.5	P0DMT3	**4.4**
F8QN51	0.7	P00622	**4.8**	P0DUN6	**8.7**

F8QN52	0.73	P00626	0.6	P0DUN7	0.4
F8QN53	0.6	P04417	0.6	P22640	0.9
O42189	0.4	P06860	0.6	P70089	0.5
O42190	0.4	P0C8M1	**2.7**	P70090	0.5
O42191	0.3	P0CAS3	0.6	P80963	0.5
O42192	0.6	P0CAS4	0.8	P82950	0.4
P00620	**7.0**	P0CAS5	0.8	P86453	0.5
P00621	**7.0**	P0CAS6	0.7	P86975	0.4
P00623	0.7	P0CAS7	**4.9**	Q2PWA3	0.6
P00624	0.7	P0DKR5	0.9	Q2YHJ4	0.7
P00625	0.6	P0DPS4	0.6	Q2YHJ8	0.7
P06859	1.2	P0DQQ0	1.1	Q2YHJ9	0.8
P0CAR9	**2.8**	P0DUN1	1.6	Q3HLQ4	0.6
P0CAS0	**3.7**	P0DUN2	**4.3**	Q6EAN6	0.8
P0DKR3	0.8	P11407	0.6	Q6H3D1	0.5
P14418	0.4	P14420	0.7	Q6H3D2	0.8
P20249	0.6	P14424	0.6	Q6H3D3	0.5
P20476	0.6	P20474	0.7	Q6H3D4	0.5
P21789	0.6	P31854	0.8	Q6H3D5	0.5
P24293	0.5	P51972	0.5	Q6H3D6	1.8
P24294	0.7	P59071	0.7	Q6JK69	0.5
P31100	0.8	P59264	0.6	Q8UVU7	0.5
P59170	0.8	P59265	0.5	Q8UVZ7	0.5
P59171	0.7	P62023	0.8	Q92152	0.7
P59172	0.7	P81458	0.6	Q9PVE3	0.4
P70088	0.7	P84674	0.7	Q9PVF3	1.2
P81243	**2.8**	P86169	0.8	Q9PVF4	0.6
P81478	0.7	P86368	0.6		
P81479	0.5	P86803	**2.8**		
P81480	0.5	P86804	**2.7**		
P82896	0.5	P86805	**4.5**		
P84651	0.6	P86806	**4.4**		
P86389	**3.0**	Q02471	0.7		
P86456	**4.9**	Q02517	0.7		
P86907	0.7	Q10755	0.8		
Q2HZ28	0.6	Q1RP78	0.7		
Q2PG83	1.2	Q1RP79	0.8		
Q2YHJ3	0.6	Q1ZY03	0.6		
Q2YHJ5	0.7	Q2PG81	0.6		
Q2YHJ6	0.7	Q2YHJ2	0.7		
Q6H3C6	0.4	Q2YHJ7	0.8		
Q6H3C7	0.7	Q6EER2	0.9		
Q6H3C8	0.7	Q6EER3	0.7		
Q6H3C9	0.5	Q6EER4	0.7		
Q6H3D0	0.6	Q6EER5	0.6		
Q7LZQ4	0.4	Q6EER6	0.6		
Q7SID6	0.4	Q6H3C5	0.6		
Q7T2R1	0.8	Q71QE8	0.7		
Q7T3S7	0.5	Q805A2	0.7		
Q7ZTA6	0.5	Q8JFG0	0.9		
Q7ZTA7	0.5	Q8JIG0	0.6		

Q7ZTA8	0.6	Q90W39	0.7
Q800C1	0.6	Q90Y77	0.6
Q800C2	0.6		
Q800C3	0.6		
Q800C4	0.7		
Q8AXY1	0.8		
Q8JIY9	0.6		
Q8QG87	0.7		
Q910A1	0.8		
Q91506	1.3		
Q92147	0.7		
Q98996	0.9		
Q9I8F8	0.6		
Q9I968	0.5		
Q9PVE9	0.5		
Q9PVF2	0.7		
Q9PWR6	0.7		
Q9YGJ7	**4.6**		

Table S6. i-face qualification and quantification contacts. Here we present the number of contacts and the nature of the residues involved in the protein-membrane interface.

IPROT CODE	Protein classification	Hydrophobic	Semipolar	Polar	Positively charged	Negatively charged	Total
A8CG78	Catalytic acidic	10	1	11	2	2	26
A8CG86	Catalytic acidic	11	1	10	3	2	27
A8CG87	Catalytic acidic	10	1	11	2	2	26
B5U6Z2	Catalytic acidic	10	1	9	3	3	26
B6CQR5	Catalytic acidic	11	1	11	2	2	27
C0HJC1	Catalytic acidic	11	1	7	4	4	27
C3W4R6	Catalytic acidic	10	1	10	3	3	27
C9DPL5	Catalytic acidic	10	0	7	7	2	26
D0UGJ0	Catalytic acidic	9	1	9	4	3	26
D6MKR0	Catalytic acidic	10	0	7	4	2	23
F8QN51	Catalytic acidic	7	1	12	5	2	27
F8QN52	Catalytic acidic	13	0	9	3	2	27
F8QN53	Catalytic acidic	7	0	11	4	2	24
O42189	Catalytic acidic	11	1	5	6	3	26
O42190	Catalytic acidic	14	1	4	6	3	28
O42191	Catalytic acidic	12	1	5	5	3	26
O42192	Catalytic acidic	11	1	8	6	2	28
P00620	Catalytic acidic	8	0	15	5	2	30
P00621	Catalytic acidic	7	0	14	4	4	29
P00623	Catalytic acidic	11	0	8	4	1	24
P00624	Catalytic acidic	11	0	9	3	2	25
P00625	Catalytic acidic	11	1	8	4	2	26
P06859	Catalytic acidic	11	1	9	6	1	28
P0CAR9	Catalytic acidic	8	2	11	6	2	29
P0CAS0	Catalytic acidic	10	2	7	5	1	25
P0DJP4	Catalytic acidic	9	1	8	6	3	27
P0DKR3	Catalytic acidic	9	1	11	1	3	25
P14418	Catalytic acidic	14	1	5	4	3	27

P20249	Catalytic acidic	11	1	7	4	3	26
P20476	Catalytic acidic	9	1	7	4	3	24
P21789	Catalytic acidic	6	1	11	7	4	29
P24293	Catalytic acidic	8	1	10	5	2	26
P24294	Catalytic acidic	11	1	10	3	1	26
P31100	Catalytic acidic	9	0	10	2	3	24
P59170	Catalytic acidic	9	1	11	6	1	28
P59171	Catalytic acidic	10	1	8	3	5	27
P59172	Catalytic acidic	7	1	10	5	1	24
P70088	Catalytic acidic	8	0	8	4	4	24
P81243	Catalytic acidic	8	1	15	3	2	29
P81478	Catalytic acidic	10	1	9	5	1	26
P81479	Catalytic acidic	11	1	7	3	3	25
P81480	Catalytic acidic	8	1	7	6	3	25
P82896	Catalytic acidic	10	1	7	5	4	27
P84651	Catalytic acidic	10	1	7	4	5	27
P86389	Catalytic acidic	8	1	11	5	3	28
P86456	Catalytic acidic	10	0	12	6	2	30
P86907	Catalytic acidic	11	0	6	5	3	25
Q2HZ28	Catalytic acidic	10	1	8	4	3	26
Q2PG83	Catalytic acidic	11	1	10	7	1	30
Q2YHJ3	Catalytic acidic	9	1	9	6	2	27
Q2YHJ5	Catalytic acidic	9	1	10	4	5	29
Q2YHJ6	Catalytic acidic	9	1	9	6	2	27
Q6H3C6	Catalytic acidic	10	0	7	5	3	25
Q6H3C7	Catalytic acidic	12	1	9	5	1	28
Q6H3C8	Catalytic acidic	8	0	8	4	4	24
Q6H3C9	Catalytic acidic	10	0	8	7	3	28
Q6H3D0	Catalytic acidic	9	1	7	4	3	24
Q7LZQ4	Catalytic acidic	13	1	5	4	3	26
Q7SID6	Catalytic acidic	9	0	7	6	3	25
Q7T2R1	Catalytic acidic	11	0	10	2	2	25
Q7T3S7	Catalytic acidic	8	1	10	6	0	25
Q7T3T5	Catalytic acidic	12	1	8	4	3	28
Q7ZTA6	Catalytic acidic	10	1	11	4	2	28
Q7ZTA7	Catalytic acidic	11	0	10	6	1	28
Q7ZTA8	Catalytic acidic	11	0	11	4	1	27
Q800C1	Catalytic acidic	11	0	10	6	1	28
Q800C2	Catalytic acidic	13	1	9	6	1	30
Q800C3	Catalytic acidic	13	0	8	7	1	29
Q800C4	Catalytic acidic	9	0	9	5	1	24
Q8AXY1	Catalytic acidic	7	1	10	4	2	24
Q8JIY9	Catalytic acidic	6	0	10	4	4	24
Q8QG87	Catalytic acidic	6	1	11	5	2	25
Q90ZZ9	Catalytic acidic	7	0	12	4	2	25
Q910A0	Catalytic acidic	12	0	11	1	1	25
Q910A1	Catalytic acidic	10	0	10	2	2	24
Q91506	Catalytic acidic	11	1	10	8	0	30
Q92147	Catalytic acidic	10	1	8	4	4	27
Q98996	Catalytic acidic	10	2	12	3	3	30
Q9I8F8	Catalytic acidic	9	1	7	5	3	25

Q9I968	Catalytic acidic	6	0	12	4	5	27
Q9PVE9	Catalytic acidic	9	0	9	5	2	25
Q9PVF0	Catalytic acidic	11	1	9	6	1	28
Q9PVF1	Catalytic acidic	11	0	9	6	2	28
Q9PVF2	Catalytic acidic	12	1	9	5	1	28
Q9PWR6	Catalytic acidic	10	1	10	2	3	26
Q9YGJ7	Catalytic acidic	8	0	13	5	2	28
Q6EER4	Catalytic acidic	9	1	9	5	4	28
A8CG82	Catalytic basic	12	1	9	5	3	30
A8CG84	Catalytic basic	8	1	7	7	6	29
A8CG89	Catalytic basic	9	1	11	5	2	28
A8CG90	Catalytic basic	8	0	11	6	1	26
C0HK16	Catalytic basic	7	1	6	8	4	26
C0HKC3	Catalytic basic	9	1	6	6	1	23
C0HKC4	Catalytic basic	10	1	6	7	1	25
C0HLF0	Catalytic basic	11	1	8	9	1	30
F8QN54	Catalytic basic	9	1	8	6	5	29
O42187	Catalytic basic	9	1	8	6	3	27
P00622	Catalytic basic	11	0	12	3	1	27
P00626	Catalytic basic	8	0	9	6	4	27
P04417	Catalytic basic	9	1	8	8	3	29
P06860	Catalytic basic	9	1	9	5	2	26
P0C8M1	Catalytic basic	9	2	6	5	2	24
P0CAS2	Catalytic basic	14	1	4	5	3	27
P0CAS3	Catalytic basic	14	1	5	6	3	29
P0CAS4	Catalytic basic	10	1	6	5	4	26
P0CAS5	Catalytic basic	13	0	5	8	2	28
P0CAS6	Catalytic basic	13	1	5	7	2	28
P0CAS7	Catalytic basic	13	0	9	5	3	30
P0CG56	Catalytic basic	12	1	6	6	3	28
P0DKR5	Catalytic basic	12	1	9	5	0	27
P0DPS4	Catalytic basic	8	1	11	3	2	25
P0DQQ0	Catalytic basic	11	2	8	4	2	27
P0DUN1	Catalytic basic	15	1	3	6	3	28
P0DUN2	Catalytic basic	9	0	8	6	2	25
P11407	Catalytic basic	5	1	9	6	4	25
P14420	Catalytic basic	12	0	9	5	0	26
P14424	Catalytic basic	10	0	8	5	4	27
P20474	Catalytic basic	7	1	7	8	6	29
P24027	Catalytic basic	11	1	5	7	2	26
P31854	Catalytic basic	7	1	14	3	1	26
P51972	Catalytic basic	10	1	6	7	1	25
P59071	Catalytic basic	7	1	7	8	6	29
P59264	Catalytic basic	9	1	9	6	2	27
P59265	Catalytic basic	11	2	9	4	2	28
P62022	Catalytic basic	13	0	6	6	3	28
P62023	Catalytic basic	13	0	6	6	3	28
P81458	Catalytic basic	8	1	10	5	3	27
P84674	Catalytic basic	11	0	5	6	5	27
P86169	Catalytic basic	13	0	6	6	3	28
P86368	Catalytic basic	10	1	6	6	5	28

P86803	Catalytic basic	6	2	11	4	3	26
P86804	Catalytic basic	5	2	11	4	2	24
P86805	Catalytic basic	13	0	8	5	3	29
P86806	Catalytic basic	13	0	8	5	2	28
Q02471	Catalytic basic	11	0	10	5	0	26
Q02517	Catalytic basic	8	1	9	6	3	27
Q10755	Catalytic basic	12	1	10	5	0	28
Q1RP78	Catalytic basic	13	1	8	5	0	27
Q1RP79	Catalytic basic	13	0	8	5	0	26
Q1ZY03	Catalytic basic	13	1	6	4	3	27
Q2YHJ2	Catalytic basic	7	1	9	6	5	28
Q2YHJ7	Catalytic basic	8	0	7	5	5	25
Q6EER2	Catalytic basic	12	0	7	4	4	27
Q6EER3	Catalytic basic	8	1	9	5	2	25
Q6EER5	Catalytic basic	11	0	7	4	4	26
Q6EER6	Catalytic basic	9	1	9	4	3	26
Q6H3C5	Catalytic basic	6	0	8	6	5	25
Q71QE8	Catalytic basic	10	1	8	4	3	26
Q805A2	Catalytic basic	9	1	9	4	4	27
Q8JFG0	Catalytic basic	11	0	9	3	0	23
Q8JIG0	Catalytic basic	8	1	8	7	3	27
Q90W39	Catalytic basic	10	0	8	5	4	27
Q90Y77	Catalytic basic	9	1	8	5	3	26
P58464	Catalytic basic	13	2	6	8	0	29
P45881	Catalytic basic	13	2	7	6	0	28
P81165	PLA2-like	13	1	7	5	0	26
O57385	PLA2-like	8	2	6	9	0	25
P04361	PLA2-like	10	0	11	9	0	30
P49121	PLA2-like	9	1	8	10	0	28
Q9I834	PLA2-like	8	3	7	11	0	29
P82950	PLA2-like	9	2	9	8	0	28
P84776	PLA2-like	11	1	8	7	1	28
P82287	PLA2-like	6	1	4	9	0	20
P48650	PLA2-like	8	2	9	4	2	25
P17935	PLA2-like	9	1	8	9	0	27
Q6H3D7	PLA2-like	9	1	6	6	2	24
I6L8L6	PLA2-like	8	2	6	10	0	26
P0DTS8	PLA2-like	7	1	4	7	0	19
P58399	PLA2-like	7	2	6	9	0	24
Q9IAT9	PLA2-like	7	1	7	11	0	26
P82114	PLA2-like	9	1	4	8	2	24
6MQD	PLA2-like	7	2	7	10	0	26
A0A1S5X	PLA2-like	10	2	5	8	0	25
A8E2V9	PLA2-like	13	2	4	9	0	28
B5U6Y4	PLA2-like	7	1	7	6	2	23
C0HKC1	PLA2-like	11	0	8	9	0	28
C0HKC2	PLA2-like	11	0	8	9	0	28
F8QN50	PLA2-like	8	1	8	9	0	26
O42188	PLA2-like	13	0	7	5	1	26
P0C616	PLA2-like	9	2	7	11	0	29
P0DJJ8	PLA2-like	12	2	6	9	0	29

P0DJJ9	PLA2-like	12	1	7	9	0	29
P0DKU1	PLA2-like	15	1	5	5	2	28
P0DMT1	PLA2-like	8	2	8	6	2	26
P0DMT2	PLA2-like	9	1	8	5	2	25
P0DMT3	PLA2-like	9	1	8	5	3	26
P0DUN6	PLA2-like	9	2	2	6	1	20
P0DUN7	PLA2-like	10	1	8	9	0	28
P0DUP2	PLA2-like	12	1	7	9	0	29
P22640	PLA2-like	8	2	5	9	1	25
P70089	PLA2-like	9	0	5	9	1	24
P70090	PLA2-like	8	2	4	10	1	25
P80963	PLA2-like	8	0	9	6	0	23
P86453	PLA2-like	6	1	8	10	0	25
P86975	PLA2-like	6	1	5	11	0	23
Q2PG81	PLA2-like	13	2	4	11	1	31
Q2PWA3	PLA2-like	9	1	4	9	0	23
Q2YHJ4	PLA2-like	7	2	5	8	1	23
Q2YHJ8	PLA2-like	9	2	2	9	1	23
Q2YHJ9	PLA2-like	9	1	3	11	1	25
Q3HLQ4	PLA2-like	16	1	8	4	1	30
Q6EAN6	PLA2-like	13	0	10	2	1	26
Q6H3D1	PLA2-like	9	1	4	8	0	22
Q6H3D2	PLA2-like	10	2	5	10	0	27
Q6H3D3	PLA2-like	9	2	4	12	0	27
Q6H3D4	PLA2-like	8	2	4	10	1	25
Q6H3D5	PLA2-like	9	2	4	11	0	26
Q6H3D6	PLA2-like	16	2	5	5	0	28
Q6JK69	PLA2-like	9	2	5	7	0	23
Q8UVU7	PLA2-like	10	0	6	9	0	25
Q8UVZ7	PLA2-like	10	1	7	8	0	26
Q92152	PLA2-like	11	1	3	9	0	24
Q9PVE3	PLA2-like	10	2	6	8	0	26
Q9PVF3	PLA2-like	10	1	4	9	0	24
Q9PVF4	PLA2-like	8	1	7	8	1	25

References

1. Fraczkiewicz, R. & Braun, W. Exact and Efficient Analytical Calculation of the Accessible Surface Areas and Their Gradients for Macromolecules. *J Comput Chem* **19**, 319333 (3193).
2. Magro, A. J., Soares, A. M., Giglio, J. R. & Fontes, M. R. M. Crystal structures of BnSP-7 and BnSP-6, two Lys49-phospholipases A2: quaternary structure and inhibition mechanism insights. *Biochem Biophys Res Commun* **311**, 713–720 (2003).
3. Dos Santos, J. I. *et al.* Structural, functional, and bioinformatics studies reveal a new snake venom homologue phospholipase A 2 class. *Proteins: Structure, Function and Bioinformatics* **79**, 61–78 (2011).
4. Zhao, K., Song, S., Lin, Z. & Znou, Y. Structure of a Basic Phospholipase A2 from Agkistrodon halys Pallas at 2.13 Å Resolution. *urn:issn:0907-4449* **54**, 510–521 (1998).
5. de Lima, L. F. G., Borges, R. J., Viviescas, M. A., Fernandes, C. A. H. & Fontes, M. R. M. Structural studies with BnSP-7 reveal an atypical oligomeric conformation compared to phospholipases A2-like toxins. *Biochimie* **142**, 11–21 (2017).
6. Salvador, G. H. M., dos Santos, J. I., Lomonte, B. & Fontes, M. R. M. Crystal structure of a phospholipase A2 from Bothrops asper venom: Insights into a new putative "myotoxic cluster". *Biochimie* **133**, 95–102 (2017).
7. Magro, A. J., Takeda, A. A. S., Soares, A. M. & Fontes, M. R. M. Structure of BthA-I complexed with p-bromophenacyl bromide: possible correlations with lack of pharmacological activity. *urn:issn:0907-4449* **61**, 1670–1677 (2005).
8. Murakami, M. T. *et al.* Inhibition of myotoxic activity of Bothrops asper myotoxin II by the anti-trypanosomal drug suramin. *J Mol Biol* **350**, 416–26 (2005).
9. Pan, Y. H., Epstein, T. M., Jain, M. K. & Bahnson, B. J. Five coplanar anion binding sites on one face of phospholipase A2: Relationship to interface binding. *Biochemistry* **40**, 609–617 (2001).

10. White, S. P., Scott, D. L., Otwinowski, Z., Gelb, M. H. & Sigler, P. B. Crystal structure of cobra-venom phospholipase A2 in a complex with a transition-state analogue. *Science (1979)* **250**, 1560–1563 (1990).
11. Scott, D. L. *et al.* Structures of Free and Inhibited Human Secretory Phospholipase A2 from Inflammatory Exudate. *Science (1979)* **254**, 1007–1010 (1991).
12. Scott, D. L., Otwinowski, Z., Gelb, M. H. & Sigler, P. B. Crystal structure of bee-venom phospholipase A2 in a complex with a transition-state analogue. *Science (1979)* **250**, 1563–1566 (1990).
13. Thunnissen, M. M. G. M. *et al.* X-ray structure of phospholipase A2 complexed with a substrate-derived inhibitor. *Nature 1990 347:6294* **347**, 689–691 (1990).
14. RCSB PDB - 4RFP: Crystal structure of a acidic PLA2 from Trimeresurus stejnegeri venom. https://www.rcsb.org/structure/4RFP.
15. Banumathi, S. *et al.* Structure of the neurotoxic complex vipoxin at 1.4 Å resolution. *Acta Crystallogr D Biol Crystallogr* **57**, 1552–1559 (2001).
16. Jasti, J., Paramasivam, M., Srinivasan, A. & Singh, T. P. Structure of an acidic phospholipase A2 from Indian saw-scaled viper (Echis carinatus) at 2.6 Å resolution reveals a novel intermolecular interaction. *urn:issn:0907-4449* **60**, 66–72 (2003).
17. Zhao, K., Zhou, Y. & Lin, Z. Structure of basic phospholipase A2 from Agkistrodon halys Pallas: Implications for its association, hemolytic and anticoagulant activities. *Toxicon* **38**, 901–916 (2000).
18. Gopalan, G., Thwin, M. M., Gopalakrishnakone, P. & Swaminathan, K. Structural and pharmacological comparison of daboiatoxin from Daboia russelli siamensis with viperotoxin F and vipoxin from other vipers. *Acta Crystallogr D Biol Crystallogr* **63**, 722–729 (2007).
19. Matsui, T. *et al.* SDS-induced oligomerization of Lys49-phospholipase A2 from snake venom. *Scientific Reports 2019 9:1* **9**, 1–8 (2019).
20. Watanabe, L., Soares, A. M., Ward, R. J., Fontes, M. R. M. & Arni, R. K. Structural insights for fatty acid binding in a Lys49-phospholipase A2: crystal structure of myotoxin II from Bothrops moojeni complexed with stearic acid. *Biochimie* **87**, 161–167 (2005).
21. Murakami, M. T., Melo, C. C., Angulo, Y., Lomonte, B. & Arni, R. K. Structure of myotoxin II, a catalytically inactive Lys49 phospholipase A2 homologue from Atropoides nummifer venom. *urn:issn:1744-3091* **62**, 423–426 (2006).
22. dos Santos, J. I., Soares, A. M. & Fontes, M. R. M. Comparative structural studies on Lys49-phospholipases A2 from Bothrops genus reveal their myotoxic site. *J Struct Biol* **167**, 106–116 (2009).
23. Jumper, J. *et al.* Highly accurate protein structure prediction with AlphaFold. *Nature 2021 596:7873* **596**, 583–589 (2021).
24. Balasubramanya, R., Chandra, V., Kaur, P. & Singh, T. P. Crystal structure of the complex of the secretory phospholipase A2 from Daboia russelli pulchella with an endogenic indole derivative, 2-carbamoylmethyl-5-propyl-octahydro-indol-7-yl-acetic acid at 1.8 Å resolution. *Biochimica et Biophysica Acta (BBA) - Proteins and Proteomics* **1752**, 177–185 (2005).
25. Arni, R. K. *et al.* Crystal Structure of Myotoxin II, a Monomeric Lys49-Phospholipase A2 Homologue Isolated from the Venom of Cerrophidion (Bothrops) godmani. *Arch Biochem Biophys* **366**, 177–182 (1999).
26. Waterhouse, A. M., Procter, J. B., Martin, D. M. A., Clamp, M. & Barton, G. J. Jalview Version 2—a multiple sequence alignment editor and analysis workbench. *Bioinformatics* **25**, 1189–1191 (2009).
